# Psychometric evaluation of a DSM-5-based semi-structured parent interview for externalizing disorders in preschool children

**DOI:** 10.1371/journal.pone.0357785

**Published:** 2026-09-15

**Authors:** Lea Teresa Kohl, Felix Oswald, Ann-Kathrin Thöne, Jana Rausch, Anja Görtz-Dorten, Christina Dose, Christopher Hautmann, Anne-Katrin Treier, Elena von Wirth, Johanna Ketter, Daria Kasperzack, Tobias Banaschewski, Daniel Brandeis, Anna Kaiser, Thomas Jans, Julia Geissler, Tanja Legenbauer, Tobias Renner, Luise Poustka, Kerstin Konrad, Michael Kölch, Stefanie Bienioschek, ESCAlife-consortium, Katja Becker, Manfred Döpfner

**Affiliations:** 1 Center for Child and Adolescent Cognitive Behavior Therapy (CEKIP), Faculty of Medicine and University Hospital Cologne, University of Cologne, Cologne, Germany; 2 Department of Child and Adolescent Psychiatry, Psychosomatics and Psychotherapy, Faculty of Medicine and University Hospital Cologne, University of Cologne, Cologne, Germany; 3 Clinical Child and Adolescent Psychology and Psychotherapy, Trier University, Trier, Germany; 4 Department of Child and Adolescent Psychiatry, Psychosomatics and Psychotherapy, University of Marburg & University Hospital Marburg (UKGM), Marburg, Germany; 5 Department of Child and Adolescent Psychiatry and Psychotherapy, Central Institute of Mental Health, Medical Faculty, Mannheim/Heidelberg University, Mannheim, Germany; 6 German Center for Mental Health (DZPG), Partner Site Mannheim-Heidelberg-Ulm, Mannheim, Germany; 7 Department of Child and Adolescent Psychiatry and Psychotherapy, University Hospital of Psychiatry Zurich, University of Zurich, Zurich, Switzerland; 8 Neuroscience Center Zurich, University and ETH Zurich, Zurich, Switzerland; 9 Department of Child and Adolescent Psychiatry, Psychosomatics and Psychotherapy, Centre of Mental Health, University Hospital of Würzburg, Würzburg, Germany; 10 LWL University Hospital Hamm for Child and Adolescent Psychiatry, Psychotherapy and Psychosomatics, Ruhr University Bochum, Hamm, Germany; 11 Department of Child and Adolescent Psychiatry, Psychosomatics and Psychotherapy, Center of Mental Health, University Hospital of Psychiatry and Psychotherapy Tübingen, Tübingen, Germany; 12 LEAD Graduate School and Research Network, University of Tübingen, Tübingen, Germany; 13 German Center for Mental Health (DZPG), Partner Site Tübingen, Tübingen, Germany; 14 Department of Child and Adolescent Psychiatry and Psychotherapy, University Medical Center Göttingen, Göttingen, Germany; 15 Department of Child and Adolescent Psychiatry, Center for Psychosocial Medicine, University Hospital Heidelberg, Heidelberg, Germany; 16 Section Child Neuropsychology, Department of Child and Adolescent Psychiatry, Psychosomatics, and Psychotherapy, Uniklinik RWTH Aachen, Aachen, Germany; 17 JARA-BRAIN Institute II, Molecular Neuroscience and Neuroimaging (INM-11), Forschungszentrum Jülich GmbH and RWTH Aachen University, Jülich, Germany; 18 Department of Child and Adolescent Psychiatry and Psychotherapy, Faculty of Health Sciences Brandenburg, Brandenburg Medical School, University Medical Center Ruppin-Brandenburg, Neuruppin, Germany; 19 Department of Child and Adolescent Psychiatry and Psychotherapy, University of Rostock, Rostock, Germany; 20 Brandenburg Medical School Theodor Fontane (MHB), Neuruppin, Germany; 21 Department of Child and Adolescent Psychiatry and Psychotherapy, University Hospital Ruppin-Brandenburg, Neuruppin, Germany; 22 Faculty of Health Sciences, Joint Faculty of the University of Potsdam, the Brandenburg University of Technology Cottbus-Senftenberg and the Brandenburg Medical School, Potsdam, Germany; 23 Center for Mind, Brain and Behavior (CMBB), University of Marburg and Justus Liebig University Giessen, Marburg, Germany; Jawaharlal Institute of Postgraduate Medical Education and Research, INDIA

## Abstract

**Objective:**

Reliable and valid diagnostic interviews for preschool-age children are needed to identify children with externalizing behavior problems requiring treatment and thus prevent adverse outcomes. This study evaluated the psychometric properties of the semi-structured *Clinical Parent Interview for Externalizing Disorders* (ILF-EXTERNAL).

**Methods:**

Data were collected from 211 children (3;0−6;11 years, 62% boys) who were screened for participation in a multicenter study on evidence-based stepped care treatments for preschool children with externalizing symptoms (ESCApreschool). The factor structure, interrater reliability, internal consistency, convergent validity, and divergent validity of the ILF-EXTERNAL were examined from a multi-rater perspective (clinicians, parents, preschool teachers).

**Results:**

A first-order factor model with four correlated factors showed acceptable model fit and allowed for straightforward interpretability. Interrater reliability estimates were fair or moderate to good [intraclass correlation coefficients(1,1) =  .52−.74]. Categorical diagnostic agreement was moderate (κ = .44−.52). The ILF-EXTERNAL scales exhibited acceptable to good internal consistencies (α ≥ .69, ω ≥ .72), except for the *ADHD Functional Impairment* scale (α = .65, ω = .68). Correlations between the ILF-EXTERNAL scale scores and the *Externalizing Problems* scale of the *Child Behavior Checklist* (CBCL/1½−5) ranged from  .38 to  .56, whereas correlations with the *Internalizing Problems* scale ranged from  .09 to  .37. Except for one subscale, all ILF-EXTERNAL subscales correlated more strongly with the *Externalizing Problems* scale than with the *Internalizing Problems* scale, providing support for convergent and divergent validity. Cross-informant correlations between clinician and parent ratings were higher (*r* = .46−.65) than between clinician and preschool teacher ratings (*r* = .05−.33).

**Conclusion:**

The ILF-EXTERNAL shows sound psychometric properties in clinically referred preschool children with ADHD and/or ODD.

## Introduction

Systematic research on the diagnostic assessment of mental health in preschool children has lagged considerably behind that in school-age children, adolescents, and adults [1]. However, longitudinal studies indicate that early psychopathology can show meaningful continuity across development and is associated with adverse outcomes later in life [2–5]. Therefore, early identification of clinically relevant symptoms is important for timely diagnostics and age-appropriate evidence-based treatment [1].

Structured clinical interviews are important diagnostic instruments that can also be used as part of the diagnostic process in preschool children. Such interviews not only represent the gold standard for diagnosing mental disorders in different age groups [6–8] but also foster the accuracy and reliability of diagnoses [9–11]. They are employed both in research and increasingly also in clinical practice as part of a comprehensive and standardized diagnostic process [7,9,11]. Furthermore, as these interviews systematically cover diagnostic criteria, they are also beneficial for clinicians in training [9–11].

Clinical interviews can be categorized into unstructured, semi-structured, and highly structured. Typically, highly structured interviews follow a categorical approach, given their purpose of establishing categorical diagnoses [9–11]. While requiring little training, they offer limited flexibility for the interviewer to explore and rate the patient’s symptoms, and usually assess symptoms dichotomously (i.e., present or absent) using closed-ended questions [9–11]. Semi-structured interviews, by contrast, allow the interviewer to seek information about symptoms, make informed judgments, and score the patient’s responses more flexibly (e.g., using Likert-type scales). This scoring format necessitates more extensive training but enables a dimensional approach by considering the severity of symptoms [9,10,12].

For the assessment of mental disorders in childhood and adolescence, both clinical parent and patient interviews are available, with the former generally used for preschool children. Preschool versions are modified and adapted for young children, with age-appropriate questions to identify symptoms typical for this age group [13]. The *NIMH Diagnostic Interview Schedule for Children Version IV* (DISC-IV) [14] is a highly structured clinical interview that was originally developed for children and adolescents. The DISC-IV follows a categorical approach and is designed to assess more than 30 psychiatric disorders. However, due to its closed-ended questions, the interview provides limited flexibility. A developmentally adapted parent version for young children, the Diagnostic Interview Schedule for Children–Young Child Version (YC-DISC-IV) [15], was developed for children aged 3–8 years based on the parent version of the DISC-IV. The YC-DISC-IV includes developmentally appropriate adaptations, such as modified wording and a shorter assessment timeframe. To date, the YC-DISC-IV does not appear to have been formally published as a finalized instrument, and published psychometric evidence remains limited to preliminary validation of the section assessing DSM-IV [15] criteria for major depression in a sample of preschool children [16]. The *Preschool Age Psychiatric Assessment* (PAPA) [17] is a structured clinical parent interview based on the *Child and Adolescent Psychiatric Assessment* (CAPA) [18], which covers a wide range of mental disorders and follows both a categorical and a dimensional approach. The PAPA was evaluated in a sample of 307 caregivers of children aged 2–5 years, who were recruited from a general pediatric clinic [19]. The test-retest reliability was found to be substantial for attention-deficit/hyperactivity disorder (ADHD; κ = .74) and conduct disorder (CD; κ = .60), and moderate for oppositional defiant disorder (ODD; κ = .57).

Regarding semi-structured clinical interviews, the *Diagnostic Infant and Preschool Assessment* (DIPA) [20] covers symptoms of thirteen disorders as well as disorder-specific functional impairment. The DIPA was evaluated in a sample of 50 caregivers of 1–6-year-old children recruited from mental health clinics specialized for this age group [20]. The test-retest reliability was found to be moderate for ADHD (inattentive type: κ = .58; hyperactive-impulsive type: κ = .42) and fair for ODD (κ = .38). Convergent validity was supported by overall moderate correlations (ADHD inattentive type: *r =* .56, ADHD hyperactive-impulsive type: *r =* .59, ODD: *r =* .55) between the DIPA scores and the corresponding symptom scales of the DSM-based *Child Behavior Checklist* (CBCL/1½−5) [20]. Moreover, the DIPA scale scores showed good to excellent internal consistency in preschool children (ADHD inattentive type: α *=* .87, ADHD hyperactive-impulsive type: α *=* .84, ADHD combined type: α *=* .91, ODD: α *=* .89) [21]. The interrater agreement for categorical diagnoses (i.e., present vs. absent) was substantial to perfect (ADHD inattentive type: κ = .75, ADHD hyperactive-impulsive type: κ = 1.00, ADHD combined type: κ = .75, ODD: κ = 1.00), and the interrater reliability (IRR) for continuous variables (i.e., number of symptoms) was almost perfect (intraclass correlation coefficient [ICC]: ADHD inattentive type: ICC = .98, ADHD hyperactive-impulsive type: κ = .98, ADHD combined type: κ = .99, ODD: κ = .98). Furthermore, the DIPA scale scores showed good convergent and adequate divergent validity [21]. Although the *Schedule for Affective Disorders and Schizophrenia for School-Age Children-Present and Lifetime Version* (K-SADS-PL) [22] does not have an explicit preschool version, Birmaher, Ehmann [23] evaluated the K-SADS-PL in a sample of 204 caregivers of 2–5-year-old children. The analyses yielded good convergent validity between the CBCL/1½−5 subscales and corresponding categorical DSM-IV diagnoses assessed using the K-SADS-PL. Additionally, ODD and ADHD diagnoses based on the K-SADS-PL showed significant correlations with the Externalizing Problems scale of the CBCL (rho = .41 and  .43). Divergent validity was supported by negligible/small correlations between the CBCL Externalizing Problems scale and anxiety disorders (rho = .15), mood disorders (rho = .19), and emotional disorders (rho = .20). Furthermore, the IRR was good to excellent (.70 ≤ κ ≤ .86) for all diagnoses [23].

Overall, existing semi-structured clinical interviews provide a reliable and valid tool for diagnosing mental disorders in preschool children. However, debate about whether mental disorders should be viewed as categorical or dimensional concepts is still ongoing [12,24–26]. Consequently, state-of-the-art assessment instruments should be sufficiently flexible to allow for both a categorical and a dimensional assessment, thus providing further information on symptom severity or subclinical symptoms, which can serve as potential prevention targets [12,24]. Moreover, assessment instruments should assess not only clinical symptoms but also functional impairment and psychological stress associated with these symptoms [27,28]. Importantly, as it is unlikely that one single informant is sufficiently privy to a child’s situation-specific behavior, e.g., at home or in preschool, a key component of best practice in evidence-based clinical child assessment is the integration of reports by multiple informants (e.g., clinicians, parents, teachers) [12,25,29–31]. Therefore, diagnostic instruments that systematically combine the assessment of different rater perspectives within the framework of a multi-informant approach should be preferred [12].

To meet the above criteria, we developed a comprehensive set of clinical parent and patient interviews, the *Interviews according to the DSM-5 Diagnostic System of Mental Disorders for Children and Adolescents* (DISYPS-ILF; German: Interview-Leitfäden zum Diagnostik-System für psychische Störungen nach DSM-5 für Kinder und Jugendliche) [32]. The DISYPS-ILF are part of the German *Diagnostic System of Mental Disorders in Children and Adolescents based on the ICD-10 and DSM-5* (DISYPS-III; German: Diagnostik-System für psychische Störungen nach ICD-10 und DSM-5 für Kinder und Jugendliche) [33]. The DISYPS-III also includes corresponding parent-, teacher-, and (for children older than 10 years) self-report questionnaires, allowing for a comparison of ratings from parents, caregivers/teachers, and patients with clinical judgments based on interviews.

One of the DISYPS-ILF interviews covers the diagnostic criteria for externalizing disorders according to the DSM-5 (see Materials and Methods for further details): the *Clinical Parent Interview for Externalizing Disorders in Children and Adolescents* (ILF-EXTERNAL; German: *Interview-Leitfaden für Externale Störungen*). Along with a categorical assessment, this interview also enables a dimensional perspective on clinical symptoms. The psychometric properties of the ILF-EXTERNAL were comprehensively evaluated in a clinical sample of children aged 6;0–11;11 years within the ESCAschool multicenter study, with the results revealing good to excellent IRR on both the item and scale level (.83 ≤ ICC(1,1) ≤  .95) and satisfactory to good internal consistencies (.60 ≤ α ≤ .86) [34]. Moreover, overall agreement on DSM-5 diagnoses was assessed using Fleiss’ kappa, which yielded substantial to almost perfect values (.38 ≤ κ ≤ .94). Please note that κ coefficients reported for the ILF-EXTERNAL reflect interrater reliability (IRR), whereas κ coefficients reported for the PAPA reflect test-retest reliability. Therefore, these coefficients should not be directly compared as indicators of the instruments‘ reliability. With regard to the factor structure of the ILF-EXTERNAL, the basic factorial configuration of the scale scores were confirmed [35]. In terms of measurement invariance, the same organization of factor and loading patterns, but not of item thresholds, was found across clinician, parent, and teacher ratings, underlining cross-situational variability in child behavior [35]. Furthermore, as functional impairment is often considered as a prerequisite for making a diagnosis, Thöne, Dose [36] examined associations between individual symptoms and global functional impairment, and found significant variations; for example, the ADHD symptom *Easily Distracted* explained approximately five times more variance than the ADHD symptom *Careless*, even though both of these symptoms originate from the ADHD inattention dimension. A recent study, which was conducted online via video chat as part of the INTEGRATE-ADHD research project (*N* = 202, age: *M* = 12.87 years, *SD* = 3.04, 28.2% female) [37], examined the psychometric properties of the ADHD section of the ILF-EXTERNAL, and found good to excellent internal consistency for the ADHD symptom scales (α = .89 to  .93) and high IRR for both the categorical and dimensional analyses (κ = .78 to  .81; ICC(1,1) =  .97 to  .98). Moreover, convergent validity was confirmed by strong correlations between the parent interview and both the German *Symptom Checklist for Attention-Deficit/Hyperactivity Disorder* (FBB-ADHS) (*r* = .79 to  .85) and the child interview (*r* = .60 to  .71). However, the extent to which these psychometric results can be replicated in a clinical sample of preschool children remains unclear.

The overall aim of this study was to evaluate the psychometric properties and factor structure of the clinical parent interview ILF-EXTERNAL in a clinical sample of 3–6-year-old preschool children with ADHD symptoms and externalizing behavior problems. This approach includes (1) testing the underlying factor structure using confirmatory factor analysis (CFA), (2) descriptive statistics for all scales, (3) internal consistencies and item-total correlations, (4) IRR, (5) overall agreement on DSM-5 diagnoses, and (6) convergent and divergent validity.

## Materials and methods

### Participants and procedure

Data collection was based on the ESCApreschool intervention study, which is part of the research consortium ESCAlife (Evidence-Based Stepped Care of ADHD along the Lifespan) and involved nine study centers located in Germany (Marburg, Aachen, Cologne, Göttingen, Hamm/Bochum, Mannheim, Neuruppin, Tübingen, Würzburg). Recruitment commenced on February 1, 2016, at the study centers in Marburg, Mannheim, Bochum, Tübingen, and Würzburg. The Cologne site initiated recruitment slightly later, on March 14, 2016. Subsequently, three additional centers, Neuruppin (June 26, 2017), Aachen (October 26, 2017), and Göttingen (November 30, 2017), also began recruitment. The final day of recruitment across all study centers was January 31, 2020. The ESCApreschool study aims to investigate the efficacy of an individualized, stepwise-intensifying treatment program based on behavioral and pharmacological interventions for 3–6-year-old preschool children with an ADHD diagnosis according to the DSM-5 or with a diagnosis of ODD plus additional substantial ADHD symptoms [38].

Families were eligible for participation in the ESCApreschool study if the children fulfilled the following inclusion criteria: (1) age between 3;0 and 6;11 years, (2) attending preschool, (3) exhibiting externalizing behavior problems, that is, either meeting DSM-5 criteria for ADHD on the ILF-EXTERNAL or meeting DSM-5 criteria for ODD on the ILF-EXTERNAL plus substantial attention or hyperactivity/ impulsivity problems (score ≥ 0.70 on either the *Inattention* or the *Hyperactivity-Impulsivity* scale of the ILF-EXTERNAL), (4) informed consent of the parents (and, where applicable, of the preschool teachers), and assent of the child. The following exclusion criteria were applied: (1) child’s IQ < 80, (2) a diagnosis of pervasive developmental disorder, schizophrenia, bipolar disorder, or severe depressive episode, (3) insufficient German-language skills of parents, (4) psychotropic medication of the child (except for ADHD medication). and (5) child’s participation in a regular intensive behavior therapy. Further details on the background and procedures are outlined in the ESCApreschool study protocol [38]. All participating parents and preschool teachers provided written informed consent and participating children gave their assent for participation.

In the present study, we used the screening dataset of the ESCApreschool study. This dataset comprised all children for whom screening data were available and whose families provided informed consent for the scientific use of their data. The eligibility criteria described above applied to participation in the ESCApreschool study and were not used as selection criteria for the present analyses. The ILF-EXTERNAL was evaluated using data from 211 children (age range 3–6 years, *M* = 5.12, *SD* = 0.84, 62% males; for more details see Table 1), including 190 who were enrolled in the ESCApreschool study [cf. 38]. The sample additionally included 21 children who did not meet all eligibility criteria of the ESCApreschool study (i.e., so-called screening negatives; an overview of violated inclusion and exclusion criteria can be found in S1 Table). These children were retained because the present analyses were based on all available screening data and were not restricted to children eligible for participation in the intervention study. Their inclusion resulted in a more heterogeneous sample, including a broader range of externalizing symptom severity and greater variability in cognitive functioning, as children with IQ scores below 80 were not excluded. Table 2 presents descriptive statistics (*M, SD*) for all ILF-EXTERNAL scales. Clinical diagnoses of ADHD and comorbid externalizing disorders were based on the results of the ILF-EXTERNAL. The ILF-EXTERNAL baseline data were collected during screening procedure of the ESCApreschool study. The screening included an assessment of eligibility for participation in the intervention study and took place during two appointments conducted no more than eight weeks apart. Interviewers typically performed the first part of the interview (assessment of ADHD and ODD symptoms) during the first appointment and the second part (assessment of CD symptoms) during the second appointment. The ILF-EXTERNAL was conducted with the parents and was recorded by audio or video. In order to assess comorbid symptoms, clinicians applied a clinical diagnostic checklist (DCL-SCREEN) from the DISYPS-III [33].

**Table 2:** Scale characteristics, Cronbach’s alpha (α), McDonald’s omega (ω), and Range of Item-Total Correlations of the ILF-EXTERNAL in the ESCApreschool Screening Dataset.

Scale	*k* (items)	α	ω	Item-total *r* (range)	Mean (*SD)*	*n*
ADHD Symptoms	18	.79	.79	.23–.47	1.69 (0.44)	205
Inattention	9	.72	.72	.31–.46	1.51 (0.51)	205
Hyperactivity-Impulsivity	9	.75	.74	.34–.54	1.88 (0.55)	205
ADHD Functional Impairment	5	.65	.68	.19–.52	1.19 (0.58)	207
ODD/CD Symptoms – Short Version	15	.83	.85	.15–.62	0.88 (0.47)	198
ODD Symptoms	8	.79	.80	.35–.63	1.27 (0.61)	198
CD Symptoms – Short Version^a^	7	.69	.73	.19–.60	0.44 (0.43)	198
DMDD^b^	5	.74	.77	.44–.59	1.06 (0.60)	143
LPE	11	.72	.73	.21–.56	0.54 (0.41)	198
ODD/CD Functional Impairment	5	.84	.73	.57–.75	0.79 (0.74)	197

ADHD = attention-deficit/hyperactivity disorder; ODD = oppositional defiant disorder; CD = conduct disorder; DMDD = disruptive mood dysregulation disorder; LPE = limited prosocial emotions; k = number of items forming a scale.

a Due to floor effects, eight items that measured aggressive and antisocial behavior were excluded from the analyses.

b Items D01 *Recurrent Temper Outbursts* and D02 *Persistently Irritable or Angry Mood* (ILF-EXTERNAL) are only available as blinded ratings.

### Measures

The measures described below were collected at several main assessment points over the course of the ESCApreschool study [38]. The present study were restricted to baseline data collected during the screening procedure, prior to the start of any intervention.

#### Clinical parent interview for externalizing disorders in children and adolescents: ILF-EXTERNAL.

The ILF-EXTERNAL [32] is part of the DISYPS-III [33], and in the present study was conducted with one primary caregiver. The interview consists of a set of items, each exploring a DSM-5 symptom criterion. In a semi-structured approach, clinicians rate each item on a 4-point Likert scale ranging from 0 (*age-typical / not at all*) to 3 (*very much*). Higher scores indicate greater symptom severity. A brief description of symptom severity is provided for each score in order to facilitate clinical judgment. Moreover, for each item, an example sentence is provided, which describes a child’s behavior representing a rating of 3 (*very much*). Item scores of 2 (*largely/considerably pronounced*) or higher are viewed as clinically relevant and as fulfilling the DSM-5 symptom criteria. The ILF-EXTERNAL covers the following DSM-5 diagnoses (including associated ICD-10 codes) from a categorical perspective: ADHD (predominantly inattentive type, predominantly hyperactive-impulsive type, combined type), ODD, disruptive mood dysregulation disorder (DMDD), and CD, with the specifier limited prosocial emotions (LPE). From the dimensional perspective, the following scale scores can be calculated by averaging the associated item scores to provide further information about the level of severity: *Inattention* (nine items), *Hyperactivity-Impulsivity* (nine items), *ADHD Symptoms* (18 items), *ADHD Functional Impairment* (five items), *ODD Symptoms* (eight items), *CD Symptoms* (15 items) *ODD/CD Symptoms* (23 items), *DMDD* (five items, three of which are also part of the *ODD Symptoms* scale), *LPE* (11 items), and *ODD/CD Functional Impairment* (five items). Scale scores are computed by averaging the associated item scores.

In the present study, two items of the *DMDD* scale (D01 *Recurrent Temper Outbursts* and D02 *Persistently Irritable or Angry Mood*) had to be retrospectively collected during the process of blinding. For this purpose, the blinded raters formed their own clinical judgment based on the audio or video recordings. Moreover, a first data check revealed considerable floor effects for some items of the ILF-EXTERNAL, with clinicians rating these items as 0 (*age-typical/not at all*) in over 90% of cases. This problem pertained to the following items assessing aggressive and antisocial symptoms, which are typically only relevant for older children: B08 *Steals with Confrontation*, B09 *Sexual Assault*, B10 *Fire Setting*, B11 *Vandalism*, B12 *Breaking in*, B13 *Stays out at Night*, B14 *Runs Away from Home Overnight*, and B15 *Truancy.* To avoid a potential bias of the results, we excluded these items from further analyses and therefore provided shortened versions of the scale *CD Symptoms – Short Version* (seven items) and the total scale *ODD/CD Symptoms* (15 items). To ensure comparability of the scales, these items were also excluded from the corresponding parent and preschool teacher symptom checklists.

#### Symptom checklist for Attention-Deficit/Hyperactivity Disorder for preschool children: FBB-ADHS-V.

The *German Symptom Checklist for Attention-Deficit/Hyperactivity Disorder for Preschool Children* (FBB-ADHS-V; German: *Fremdbeurteilungsbogen für Vorschulkinder mit Aufmerksamkeitsdefizit-/Hyperaktivitätsstörungen*) is a standardized questionnaire based on the criteria outlined in the ICD-10 and DSM-5 diagnostic manuals. It is part of the DISYPS-III assessment system [33] and can be completed by parents or preschool teachers. The FBB-ADHS-V consists of 19 items which form identical ADHD scales to those in the ILF-EXTERNAL, and an additional six items assessing functional impairment and psychological distress associated with the ADHD symptomatology. All items are rated on a 4-point Likert scale ranging from 0 *(not at all)* to 3 *(very much)*. In both a clinical sample (*N* = 187) and a representative sample (*N* = 521), the FBB-ADHS-V scale scores rated by parents of preschool children have demonstrated sound psychometric properties in terms of reliability (.87 ≤ α ≤ .92 [clinical sample];  .88 ≤ α ≤ .94 [representative sample]) and factorial validity [33,39]. The FBB-ADHS-V scale scores rated by preschool teachers have likewise demonstrated good internal consistencies (.89 ≤ α ≤ .95 [clinical sample];  .89 ≤ α ≤ .93 [representative sample]) and factorial validity [33,39]. In the present analyses, we included the scales *ADHD Inattention*, *ADHD Hyperactivity-Impulsivity*, *ADHD Symptoms*, and *ADHD Functional Impairment*.

#### Symptom checklists for disruptive behavior disorders: FBB-SSV.

The German *Symptom Checklist for Disruptive Behavior Disorders* (FBB-SSV; German: *Fremdbeurteilungsbogen für Störungen des Sozialverhaltens*) is a standardized questionnaire based on the criteria outlined in the ICD-10 and DSM-5 diagnostic manuals. This symptom checklist is part of the DISYPS-III [33] and can be completed by parents or preschool teachers. The FBB-SSV has the same structure and assessment as the FBB-ADHS-V (see above), and encompasses 46 items, which also form disruptive behavior scales identical to those in the ILF-EXTERNAL, and an additional five items assessing functional impairment and psychological distress associated with the disruptive behavior symptomatology as well as 12 items assessing the child’s competencies. In a clinical sample (*N* = 596) as well as a community sample (*N* = 720) of school-age children, the parent-rated FBB-SSV scale scores showed acceptable to good psychometric properties with regard to reliability (.71 ≤ α ≤ .90 [community sample];  .69 ≤ α ≤ .90 [clinical sample]), as well as factorial validity and diagnostic accuracy [40]. In a sample of N = 160 preschool children (n = 80 clinical group; n = 80 matched control group), Korsch and Petermann [41] analyzed the agreement between parents’ and preschool teachers’ ratings of children’s behavior. The authors found that correlations between parent and preschool teacher ratings for the FBB-SSV (total score) were negligible in both groups (.17 ≤ rho ≤ .18), and that parent ratings were significantly higher (*p* = .001) than preschool teacher ratings [41]. In the present study, we used the scales *ODD Symptoms, CD Symptoms – Short Version, ODD/CD Symptoms – Short Version, DMDD, LPE*, and *ODD/CD Functional Impairment*.

#### ASEBA preschool-age forms & profiles: CBCL/1½−5.

To examine convergent and divergent validity, we used information from the German version of the *Child Behavior Checklist for Children Aged 1.5–5* (CBCL/1½−5) [42]. Originally developed by the Achenbach group [25], the German CBCL/1½−5 is a broadband questionnaire comprising 100 items developed to assess behavioral and emotional problems in preschool children. Items are rated on a 3-point scale ranging from 0 (*not true*), to 2 (*very true or often true*). The items form seven syndrome scales and three broadband scales (*Externalizing Problems, Internalizing Problems, Total Problems*). In a community sample of *N* = 850 preschool children, the CBCL/1½−5 syndrome scale scores demonstrated poor to good internal consistencies (.59 ≤ α ≤ .89), and the broadband scales demonstrated good internal consistencies (*Externalizing Problems:* α = .90*, Internalizing Problems* α = .86*, Total Problems* α = .95) as well as factorial validity [42]. Similar psychometric properties for the CBCL/1½−5 syndrome scale scores (.49 ≤ α ≤ .89) and the broadband scales (*Externalizing Problems:* α = .90*, Internalizing Problems* α = .82*, Total Problems* α = .93) were found in a sample of *N* = 291 preschool children with elevated behavior problems [42]. In the present study, the summary scores of the seven syndrome scales and the *Internalizing Problems* and *Externalizing Problems* scales were used.

### Subsample for the analysis of interrater reliability

To assess IRR, a subset of 129 interview recordings of the ILF-EXTERNAL conducted at baseline was randomly drawn (see Table 1 for the characteristics of this subsample). To be included in this subset, an audio or video recording for both the ADHD and ODD/CD parts of the ILF-EXTERNAL had to be available, with sufficient audio quality.

**Table 1:** Sample characteristics.

	Subsample for the analysis of interrater reliability (*n* = 129)	Total sample (*N* = 211)
Age (years): mean *(SD)*	5.15 (0.85)	5.12 (0.84)
Male: *n* (%)	103 (79.8)	170 (80.6)
Diagnosis: *n* (%)		
no ADHD diagnosis	18 (14.01)	36 (17.5)
ADHD – combined type	43 (33.6)	62 (30.1)
ADHD – predominantly inattentive type	14 (10.9)	23 (11.2)
ADHD – predominantly hyperactive-impulsive type	53 (41.4)	85 (41.3)
Comorbid internalizing disorders: *n* (%)		
Anxiety	10 (8.1)	16 (8.6)
Depression	1 (0.8)	1 (0.5)
Obsessive-compulsive disorder	3 (2.4)	3 (1.6)
Comorbid externalizing disorders: *n* (%)		
Oppositional defiant disorder	74 (57.8)	109 (52.7)
Conduct disorder	12 (9.3)	17 (8.5)
Comorbid other disorders: *n* (%)		
Tic disorder	8 (6.5)	8 (4.3)
Autism spectrum or social communication disorder	0 (0.0)	1 (0.5)
ADHD medication: *n* (%)	0 (0.0)	0 (0.0)
German as parents’ primary language: *n* (%)	123 (95.3)	190 (95.0)
Parents’ highest educational attainment: *n* (%)^a^		
Pre-primary and primary education	0 (0.0)	0 (0.0)
Lower secondary education	0 (0.0)	0 (0.0)
Upper and post-secondary education	38 (39.6)	60 (39.7)
Tertiary education	57 (59.4)	89 (58.9)
Others (foreign qualification)	1 (1.0)	2 (1.3)

The diagnoses of ADHD and comorbid externalizing disorders were established using the semi-structured clinical parent interview ILF-EXTERNAL, which was conducted with the parents/caregivers. Additional symptoms of comorbidity were evaluated using a clinical diagnostic checklist (DCL-SCREEN)

ADHD = attention-deficit/hyperactivity disorder

^a^The classification of parents’ highest educational attainment is based on the International Standard Classification of Education [43].

### Interview training

All interviewers involved in the recruitment of patients for the ESCApreschool study were trained psychologists or educationalists with a Master’s degree, PhD students, or students undergoing child and adolescent psychotherapy/psychiatry training. During the ESCApreschool study, all of the interviewers received extensive, standardized training on administering and scoring the ILF-EXTERNAL prior to conducting the interviews. The training included watching a practice video, observing interviews conducted by others, and carrying out a minimum of three interviews under supervision. For the blinded ratings (used to determine interrater reliability in the present study), seven employees who were not previously affiliated with the study assessments underwent the same training, and additionally scored three practice videos with a limited margin of error to an established gold standard. If interviewers experienced any difficulty in carrying out assessments using the ILF-EXTERNAL, they were advised to consult their supervisor.

### Data analytic plan

All statistical analyses were performed using R [44]. An initial check of the data did not show any considerable floor or ceiling effects of the ILF-EXTERNAL item frequencies (with the exception of the items that had been previously excluded). If over 10% of the items from a particular scale were missing, we did not compute this scale for the respective participant to avoid a possible bias of the results [45]. We likewise applied this listwise exclusion criterion to the scales of the parent and preschool teacher questionnaire data.

#### Confirmatory factor analysis.

Confirmatory factor analysis (CFA) was used to investigate the factor structure of the ILF-EXTERNAL. Specifically, we tested a unidimensional model, a first-order correlated factors model with four factors (ADHD Inattention, ADHD Hyperactivity-Impulsivity, ODD, CD), a first-order correlated factors model with two factors (ADHD, ODD/CD), and a higher-order factor model with one second-order factor (externalizing symptoms) as well as four first-order factors (ADHD Inattention, ADHD Hyperactivity-Impulsivity, ODD, CD). The selection of the tested models was informed by previous psychometric evaluation of the ILF-EXTERNAL in school-age children [35], in which several theoretically plausible factor structures were examined. For the present preschool sample, we adapted the tested models to the specific aims of the study, with a primary focus on theoretically relevant and clinically interpretable DSM-5-based symptom domains in preschool children. For parameter estimation (ordinal data), we used the weighted least squares means and variance adjusted estimator (WLSMV). To evaluate the model fit of the tested models, the following parameters were applied: χ^2^ test of exact model fit, the comparative fit index (CFI), the Tucker–Lewis index (TLI), the root mean square error of approximation (RMSEA) with 90% confidence intervals, and the standardized root mean square residual (SRMR). Model fit was considered acceptable if RMSEA and SRMR were ≤ .08 and as good if CFI and TLI were ≥ .95 and RMSEA and SRMR were ≤ .05 [46,47]. As the χ^2^ test is overly sensitive to sample size, greater weight was given to CFI, TLI, RMSEA, and SRMR. However, the above-mentioned cut-off values for these fit indices should only be interpreted as rough guidelines, and it is important to additionally consider the statistical and theoretical conformity of the factor models. Furthermore, to evaluate whether one model provides a better fit than another model, the Akaike information criterion (AIC) and the Bayesian information criterion (BIC) were calculated using maximum likelihood estimation with robust standard errors (MLR).

#### Scale characteristics.

In addition to descriptive statistics (mean scores, standard deviations) for all of the ILF-EXTERNAL scales, we calculated Cronbach’s alpha (α) and McDonald’s omega (ω), with values ≥ .70 indicating acceptable internal consistency [48]. Furthermore, corrected item-total correlations were calculated, with values ≥ .30 considered as acceptable [49].

#### Interrater reliability.

To assess interrater reliability between the original interviewers (semi-blinded) and the independent blind raters, we calculated the ICC [50,51], which is one of the most commonly used metrics to assess interrater reliability for continuous data [52–54]. It should be noted that different formulae exist, which each involve distinct assumptions about their calculations and thus lead to different interpretations [53]. In the present study, we calculated the ICC one-way random-effects, absolute agreement model for single rater/measurements (ICC (1,1)) and for measures based on a mean-rating ICC(1,*k*) with their 95% confidence intervals (CIs). We chose this ICC one-way model because the physical distance between the study centers prevented the same interviewer from assessing all participants, which would have qualified for two-way measurement models [53]. Furthermore, in our view, the single-rater/measurements model ICC(1,1) is more appropriate than average measures, as the clinical outcome of the ILF-EXTERNAL should be based on one clinician rather than on information averaged across multiple clinicians [53]. Nevertheless, to ensure comparability of our results with other studies, we also present average measurements ICC(1,k). For the interpretation of ICC coefficients, different benchmarks are commonly cited. For instance, Cicchetti [55] provided the following guidelines: poor ≤ .40; fair = .41–.59; good = .60–.74; and excellent ≥ .75. By contrast, Koo and Li [53] proposed more stringent guidelines: poor ≤ .50; moderate = .51–.75; good = .76–.90; and excellent ≥ .91. Accordingly, the results are interpreted according to the proposed guidelines both by Koo and Li [53] and Cicchetti [55].

Cohen’s kappa was used to assess overall agreement on DSM-5 diagnoses [56]. This is a statistical measure to assess agreement between two raters (i.e., the interviewer and one rater) regarding categorical variables (i.e., whether a disorder is present or absent). We derived the presence or absence of a disorder from the raw interview item scores. For example, a diagnosis of ADHD: predominantly inattentive type was seen as present if at least six items from the *ADHD Inattention* scale and fewer than six items from the *ADHD Hyperactivity-Impulsivity* scale were scored with 2 or above. We calculated Cohen’s kappa between the original interviewer and the blinded rater. Landis and Koch [57] suggested the following benchmarks for the interpretation of kappa values: slight < .20; fair = .21 –  .40; moderate = .41 –  .60; substantial = .61 –  .80; almost perfect agreement > .81.

#### Convergent and divergent validity.

In order to evaluate convergent validity between clinical judgment and parent or preschool teacher ratings, Pearson product-moment correlations (*r*) were calculated between all ILF-EXTERNAL scales and the corresponding scales of the parent and preschool teacher forms (FBB-ADHS-V; FBB-SSV). We used paired samples two-sided *t*-tests for group comparisons between the average scores of the ILF-EXTERNAL scales and the corresponding scales of the parent or preschool teacher forms.

For further assessment of convergent and divergent validity, Pearson product-moment correlations were calculated between the ILF-EXTERNAL scales and the seven syndrome scales as well as the Externalizing Problems and Internalizing Problems scales from the CBCL/1½−5. The R *cocor* package and Steiger’s test [58] were used to compare the magnitude of two dependent correlations. In particular, we were interested in whether the correlation coefficient between a particular ILF-EXTERNAL scale (e.g., *Inattention*) and the CBCL/1½−5 *Externalizing Problems* scale differed significantly from that between the same ILF-EXTERNAL scale and the *Internalizing Problems* scale. A priori, stronger associations were expected between ILF-EXTERNAL scores and measures of externalizing symptoms than with measures of internalizing symptoms, reflecting the hypothesized construct specificity of the instrument.

### Ethical considerations

The ESCApreschool study was approved by the Ethics Committee of the Medical Faculty of the Phillips University of Marburg, Germany (No. 87/ 15) and by all local Ethics Committees of the participating centers in Germany: Faculty of Medicine of the Ruhr-University Bochum (No. 15–5523), Medical Faculty of the University of Cologne (No. 15–335), Ethics Committee of the State Chamber of Medicine (Landesärztekammer) Baden-Württemberg (No. B-F-2015–080), Medical Faculty of the University of Würzburg (No. 254/15_z), Faculty of Medicine of the Eberhard Karls University Tübingen (No. 728/2015BO2), Medical Faculty of the University of Aachen (No. EK 204/17), Medical Faculty of the University of Göttingen (No. 4/3/17), and the Brandenburg Medical School Theodor Fontane (Z-01–20170208). To participate in the study, both parents/guardians are informed about the study procedures and goals, and provide written informed consent. Additionally, the preschool child has to give his/her assent for participation.

## Results

### Confirmatory factor analysis

**Fig 1:**
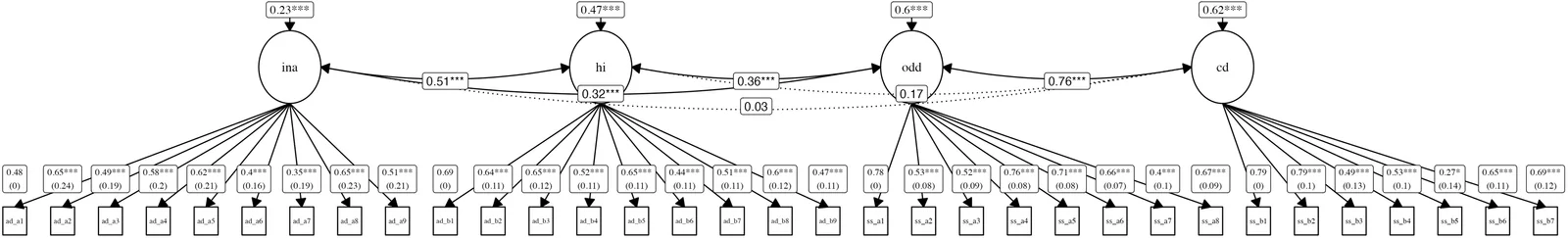
Standardized factor loadings and covariance structure of the first-order CFA with four correlated factors. Factor descriptions: ina = Inattention; hi = Hyperactivity-Impulsivity; odd = Oppositional Defiant Disorder; cd = Conduct Disorder. Item descriptions: ad_a1 = Careless; ad_a2 = Difficulties Sustaining Attention; ad_a3 = Does not Listen; ad_a4 = Does not Finish Work; ad_a5 = Organizational Skills; ad_a6 = Concentration; ad_a7 = Loses Things; ad_a8 = Easily Distracted; ad_a9 = Forgetful; ad_b1 = Fidgets; ad_b2 = Leaves Seat; ad_b3 = Runs, Climbs; ad_b4 = Difficulties Playing Quietly; ad_b5 = Driven, on the go; ad_b6 = Blurts out Answers; ad_b7 = Waits for Turn; ad_b8 = Interrupts, Intrudes; ad_b9 = Talks Excessively; ss_a1 = Loses Temper; ss_a2 = Touchy, Easily Annoyed; ss_a3 = Angry, Resentful; ss_a4 = Argues with Adults; ss_a5 = Refuses to Comply with Requests; ss_a6 = Annoys; ss_a7 = Blames Others; ss_a8 = Spiteful, Vindictive; ss_b1 = Physical Fights; ss_b2 = Bullies, Threatens, or Intimidates; ss_b3 = Cruel to Animals; ss_b4 = Lies; ss_b5 = Steals without Confrontation; ss_b6 = Uses Weapons in Fight; ss_b7 = Cruel to People. Due to floor effects, eight items that measured aggressive and antisocial behavior were excluded from the analyses. *p < .05, ***p < .001.

The goodness-of-fit indices for the ILF-EXTERNAL factor models are summarized in Table 3. For our unidimensional model, the model fit indices did not meet the aforementioned cut-off criteria, resulting in a relatively poor model fit. However, the model with four correlated factors showed a generally acceptable model fit (RMSEA = .04, SRMR = .09), all standardized CFA factor loadings were significant, and λ ≥ 0.27 (Fig 1). Examining the factor correlations of the first-order CFA model with four correlated factors revealed moderate to high significant correlations between the Inattention and the Hyperactivity-Impulsivity factors (*r* = .51), and between ODD and CD (*r* = .76). ODD also showed a significant but low correlation with Inattention (*r* = .32) and with Hyperactivity-Impulsivity (*r* = .36). The correlations between CD and Inattention and between CD and Hyperactivity-Impulsivity remained non-significant. All remaining factor models tested revealed model fit indices that were inferior to those of the model with four correlated factors and did not meet the criteria for an acceptable fit. Although the bifactor CFA showed slightly lower AIC than the first-order CFA model with four correlated factors (Δ = 9.27), the latter showed a lower BIC (Δ = 79.23), indicating a more parsimonious solution. Given its acceptable model fit and direct correspondence to the DSM-5-based symptom domains assessed by the ILF-EXTERNAL, the first-order model with four correlated factors was considered the most appropriate representation of the data. Additional findings regarding the factor loadings and (co-)variance structure of the remaining factor models can be found in the supporting information (S1-S4 Figs).

**Table 3:** Goodness-of-fit statistics and information criteria for the CFA models.

Model^a^	*χ* ^2^	*df*	CFI	TLI	RMSEA [90% CI]	SRMR	AIC^b^	BIC^b^
Unidimensional CFA	1.202.293*	495	.595	.568	.086 [.079, .092]	.136	16174.11	16390.47
First-order CFA (4 factors)	646.952*	489	.902	.902	.041 [.032, .049]	.090	15731.95	15967.98
First-order CFA (2 factors)	773.497*	494	.829	.829	.054 [.046, .061]	.103	15880.06	16099.70
Second-order CFA	755.126*	491	.849	.837	.053 [.045, 0.060]	.103	15750.36	15979.83
Bifactor CFA	694.499*	462	.867	.848	.051 [.043, 0.058]	.094	15722.68	16047.21

CFA = confirmatory factor analysis; *df* = degrees of freedom; CFI = comparative fit index; TLI = Tucker-Lewis index; RMSEA = root mean square error of approximation; CI = confidence interval; SRMR = standardized root mean square residual; AIC = Akaike information criterion; BIC = Bayesian information criterion.

^a^Due to floor effects, eight items that measured aggressive and antisocial behavior were excluded from the analyses.

^b^All factor models were calculated using the *weighted least squares means and variance adjusted* (WLSMV) estimator for categorical indicators. Information criteria were calculated using *maximum likelihood estimation with robust standard errors* (MLR) for continuous indicators.

*p < .001.

### Scale characteristics

The mean scores, standard deviations, internal consistencies (Cronbach’s alpha), and range of the item-total correlations for all of the ILF-EXTERNAL scales are summarized in Table 2. The lowest mean score emerged for the scale *CD Symptoms – Short Version* (*M* = 0.44, *SD* = 0.43), and the highest mean score was observed for the scale *Hyperactivity-Impulsivity* (*M* = 1.88, *SD* = 0.55). Cronbach’s alpha coefficients for the ILF-EXTERNAL symptom scales were acceptable to good, apart from the scale *CD Symptoms – Short Version* (α = .69), though the stricter McDonald’s Omega was still within the acceptable range (ω = .73). Regarding the scales assessing *Functional Impairment*, internal consistency was low for ADHD (α = .65) and good for ODD/CD (α = .84). Item-total correlations were largely satisfactory (.23 ≤ *r*_it_ ≤ .63), although there were some exceptions, with the following items showing item-total correlations below *r*_it_ = .30: ADHD items: A06 *Concentration*, A07 *Loses Things*. ODD/CD items: B05 *Steals Without Confrontation*, C03a *Indifferent of Poor Performance*, C3b *Avoids Effort*, C03c *Blames Others for Poor Performance*, C04a *Shallow / Deficient*, C04c *Manipulates*, C04d *Inconsistent Affect*. However, the Cronbach’s alpha of the respective scales did not noticeably change if any of these items were excluded.

### Interrater reliability

Table 4 provides the IRR of the ILF-EXTERNAL ADHD and ODD scales, based on the ICC one-way random-effects, absolute agreement model for single ICC(1,1) and average measures ICC(1,2) as well as their respective 95% confidence intervals. Regarding the scales *ADHD Symptoms* [ICC(1,1) =  .52] and *ADHD Inattention* [ICC(1,1) =  .58], the ICC(1,1) indicate fair or moderate IRR according to Cicchetti [55] or Koo and Li [53]. For the *ADHD Hyperactivity-Impulsivity* scale [ICC(1,1) =  .60] and the *ODD Symptoms* scale [ICC(1,1) =  .74], the IRR was good [55] or rather moderate [53].The ICC(1,2) of the *ADHD Symptoms* scale [ICC(1,2) =  .68] and the *ADHD Inattention* scale [ICC(1,2) =  .73] indicated good IRR according to Cicchetti [55] and moderate IRR according to Koo and Li [53]. For the *ADHD Hyperactivity-Impulsivity* scale [ICC(1,2) =  .75] and the *ODD Symptoms* scale [ICC(1,2) =  .85], the IRR was excellent [55] or rather good [53].

**Table 4:** Interrater reliability of the ILF-EXTERNAL scales.

Scale	ICC(1,1)	95% CI	ICC(1,2)	95% CI	*n*
ADHD symptoms	.52	[.38, .63]	.68	[.55, .78]	129
Inattention	.58	[.45, .68]	.73	[.62, .81]	129
Hyperactivity-Impulsivity	.60	[.47, .70]	.75	[.64, .82]	129
ODD symptoms	.74	[.65, .81]	.85	[.79, .89]	129

ADHD = attention-deficit/hyperactivity disorder; CI = confidence interval; ICC = intraclass correlation; ICC(1,1) = one-way random-effects, absolute agreement model for single rater/measurements; ICC(1,2) = one-way random effects, absolute agreement model based on a mean rating.

### Agreement on DSM-5 diagnoses

Table 5 presents the overall agreement regarding DSM-5 diagnoses assessed using Cohen’s kappa values and their corresponding 95% confidence intervals. According to the benchmarks of Landis and Koch [57], agreement on categorical diagnoses was moderate (ADHD combined type & ADHD inattentive type: κ = .44; ADHD hyperactive-impulsive type: κ = .52; ODD: κ = .52).

**Table 5:** Interrater agreement on DSM-5 diagnoses in the subsample (n = 129) for the analysis of interrater reliability.

DSM-5 Diagnosis	Cohens Kappa	95% CI	Base rate *n*
ADHD: combined type	.44	[.28, .60]	44
ADHD: predominantly inattentive type	.44	[.17, .71]	15
ADHD: predominantly hyperactive- impulsive type	.52	[.38, .66]	53
Oppositional defiant disorder	.52	[.39, .65]	75

ADHD *=* attention-deficit/hyperactivity disorder; CI = confidence interval. The presence of a disorder was derived from the raw interview item scores by symptom counts. 17 cases had no ADHD diagnosis.

### Convergent and divergent validity

Table 6 presents the correlations between the ILF-EXTERNAL scales and the corresponding scales of the parent forms (FBB-ADHS-V; FBB-SSV). Pearson correlations were moderate to high and significant (.46 ≤ *r* ≤ .69, *p* < .001), indicating acceptable to good convergent validity between clinical judgment and parent ratings. Ratings on the ILF-EXTERNAL scales *ADHD Functional Impairment, DMDD, LPE, and ODD/CD Functional Impairment* differed significantly from ratings on the corresponding scales of the parent forms (*p* ≤ .01), with higher mean scale scores on the parent forms for the *LPE, ODD/CD Functional Impairment,* ADHD *Functional Impairment*, and *DMDD* scales compared to clinician ratings.

**Table 6:** Comparisons of the ILF-EXTERNAL scales and the corresponding parent forms (FBB-ADHS-V and FBB-SSV).

Scale	ILF- EXTERNAL: Mean *(SD)*	FBB (parents): Mean *(SD)*	*r*	Paired samples *t*-test	*p*
ADHD Symptoms	1.69 (0.44)	1.70 (0.55)	.57	t(185) = 0.26	.798
Inattention	1.51 (0.51)	1.54 (0.57)	.46	t(185) = −0.18	.856
Hyperactivity- Impulsivity	1.88 (0.55)	1.85 (0.65)	.60	t(185) = 1.04	.299
ADHD Functional Impairment	1.19 (0.58)	1.35 (0.70)	.52	t(166) = −2.61	.010
ODD/CD Symptoms Short Version^a^	0.97 (0.50)	0.98 (0.50)	.69	t(181) = −0.31	.760
ODD Symptoms	1.27 (0.61)	1.36 (0.65)	.65	t(181) = −2.11	.036
CD Symptoms Short Version^a^	0.49 (0.46)	0.49 (0.42)	.62	t(181) = 0.35	.729
DMDD^b^	1.06 (0.60)	1.22 (0.70)	.64	t(135) = −3.65	<.001
LPE	0.54 (0.41)	0.62 (0.42)	.52	t(181) = −2.93	.004
ODD/CD Functional Impairment	0.79 (0.74)	1.12 (0.76)	.65	t(142) = −6.00	<.001

ILF-EXTERNAL = Clinical Parent Interview for Diagnosing Externalizing Disorders in Children and Adolescents; FBB-ADHS-V = Symptom Checklist for Attention-Deficit/Hyperactivity Disorder for Preschool Children; FBB-SSV = Symptom Checklist for Disruptive Behavior Disorders; ADHD = attention-deficit/hyperactivity disorder; ODD = oppositional defiant disorder; CD = conduct disorder; DMDD = disruptive mood dysregulation disorder; LPE = limited prosocial emotions. All bivariate correlations are significant at *p* < .001 (unadj.).

^a^Due to floor effects, eight items that measured aggressive and antisocial behavior were excluded from the analyses.

^b^Items D01 *Recurrent Temper Outbursts* and D02 *Persistently Irritable or Angry Mood* (ILF-EXTERNAL) are only available as blinded ratings.

Table 7 presents the correlations between the ILF-EXTERNAL scales and the corresponding scales of the proxy rating forms completed by preschool teachers (FBB-ADHS-V; FBB-SSV). Pearson correlations were negligible to moderate (.05 ≤ *r* ≤ .33). Furthermore, the mean scale scores on the preschool teacher ratings differed significantly (*p* < .01) from the corresponding clinician ratings (with the exception of *Inattention, ODD/CD Symptoms-Short Version*, and *ODD Symptoms-Short Version*, *DMDD*). Overall, clinicians’ mean scale scores were significantly higher for ADHD hyperactivity-impulsivity symptoms (*p* < .01), whereas preschool teachers provided higher ratings for the majority of the disruptive behavior symptoms (*p* < .01).

**Table 7:** Comparisons of the ILF-EXTERNAL scales and the corresponding preschool teacher forms (FBB-ADHS-V and FBB-SSV).

Scale	ILF- EXTERNAL: Mean *(SD)*	FBB (preschool teacher): Mean *(SD)*	*r*	Paired samples *t*-test	*p*
ADHD Symptoms	1.69 (0.44)	1.55 (0.69)	.14	t(120) = 2.89	.005
Inattention	1.51 (0.51)	1.50 (0.73)	.13	t(120) = 0.70	.484
Hyperactivity- Impulsivity	1.88 (0.55)	1.59 (0.77)	.19*	t(120) = 4.54	<.001
ADHD Functional Impairment	1.19 (0.58)	1.55 (0.77)	.30**	t(103) = −3.57	.001
ODD/CD Symptoms Short Version^a^	0.97 (0.50)	1.04 (0.72)	.30***	t(115) = −0.86	.393
ODD Symptoms	1.27 (0.61)	1.21 (0.80)	.27**	t(115) = 1.09	.277
CD Symptoms Short Version^a^	0.49 (0.46)	0.70 (0.68)	.33***	t(115) = −3.57	.001
DMDD^b^	1.06 (0.60)	0.98 (0.85)	.15	t(86) = 1.03	.305
LPE	0.54 (0.41)	0.89 (0.67)	.05	t(115) = −4.62	<.001
ODD/CD Functional Impairment	0.79 (0.74)	1.44 (0.88)	.22*	t(89) = −5.25	<.001

ILF-EXTERNAL = Clinical Parent Interview for Diagnosing Externalizing Disorders in Children and Adolescents; FBB-ADHS-V = Symptom Checklist for Attention-Deficit/Hyperactivity Disorder for Preschool Children; FBB-SSV = Symptom Checklist for Disruptive Behavior Disorders; ADHD = attention-deficit/hyperactivity disorder; ODD = oppositional defiant disorder; CD = conduct disorder; DMDD = disruptive mood dysregulation disorder; LPE = limited prosocial emotions. *p < .05; **p < .01; ***p < .001 (unadj.).

^a^Due to floor effects, eight items that measured aggressive and antisocial behavior were excluded from the analyses.

^b^Items D01 *Recurrent Temper Outbursts* and D02 *Persistently Irritable or Angry Mood* (ILF-EXTERNAL) are only available as blinded ratings.

Table 8 provides a summary of the Pearson correlations of the ILF-EXTERNAL scales with the seven CBCL/1½−5 syndrome scales and with the CBCL/1½−5 broadband scales *Externalizing Problems* and *Internalizing Problems*. On the whole, the ILF-EXTERNAL scales showed significant, moderate to high correlations with the CBCL *Externalizing Problems* scale (.38 ≤ *r* ≤ .56). Comparison of the dependent correlations using Steiger’s test revealed that all ILF-EXTERNAL scales, except the *Inattention Symptoms* subscale, showed significantly stronger associations with the CBCL *Externalizing Problems* scale than with the *Internalizing Problems* scale (.001 ≤ *p* ≤ .018). Moreover, the CBCL *Externalizing Problems* scale showed the highest correlations with the ILF-EXTERNAL scales *ODD/CD Symptoms – Short Version* (*r* = .40) and *CD Symptoms – Short Version* (*r* = .56).

**Table 8:** Correlations of the ILF-EXTERNAL scales and the child behavior checklist (CBCL/1½−5)scales.

	CBCL/1½−5										
Scale	Emot. Reactive	Anxious/ Depressed	Somatic complaints	Social withdrawal	Sleep Probl.	Attention Probl.	Aggressive behavior	Ext. Probl.	Int. Probl.	Ext. vs. Int.	
	*r* [95% CI]	*r* [95% CI]	*r* [95% CI]	*r* [95% CI]	*r* [95% CI]	*r* [95% CI]	*r* [95% CI]	*r* [95% CI]	*r* [95% CI]	*z*	*p*
ADHD Symptoms	.38 [.25, .50]	.30 [.17, .43]	.17 [.02, .30]	.25 [.11, .38]	.18 [.04, .32]	.58 [.47, .66]	.42 [.30, .54]	.51 [.39, .61]	.37 [.24, .49]	2.49	.010
Inattention	.30 [.17, .43]	.28 [.15, .41]	.12 [-.03, .26]	.26 [.12, .39]	.14 [.00, .28]	.45 [.33, .56]	.31 [.18, .44]	.38 [.25, .50]	.32 [.18, .44]	1.01	.310
Hyperactivity- Impulsivity	.32 [.19, .44]	.22 [.08, .35]	.15 [.01, .29]	.16 [.02, .30]	.15 [.01, .29]	.49 [.37, .59]	.38 [.25, .50]	.45 [.33, .56]	.29 [.15, .41]	2.73	.006
ODD/CD Symptoms – Short Version ^a^	.20 [.06,.33]	.19 [.05, .33]	.10 [-.02, .24]	.13 [-.02, .27]	.10 [-.05, .24]	.11 [-.04, .25]	.62 [.52, .70]	.56 [.45, .65]	.21 [.06, .34]	6.06	< .001
ODD Symptoms	.25 [.11, .38]	.20 [.06, .33]	.12 [-.02, .26]	.15 [.00, .29]	.12 [.03, .26]	.11 [-.03, .25]	.62 [.52, .70]	.56 [.45, .65]	.24 [.10, .37]	5.61	< .001
CD Symptoms – Short Version ^a^	.07 [-.08, .21]	.12 [-.03, .26]	.03 [-.12, .17]	.06 [-.09, .20]	.03 [-.11, .18]	.06 [.08, .21]	.44 [.31, .55]	.40 [.27, .51]	.09 [-.06, .23]	5.03	< .001
Disruptive Mood Dysregulation	.34 [.19, .48]	.29 [.12, .43]	.16 [-.01, .26]	.10 [-.07, .26]	.20 [.03, .35]	−.02 [-.18, .15]	.55 [.42, .65]	.47 [.33, .59]	.31 [.15, .45]	2.37	.018
Limited Prosocial Emotions	.20 [.06, .33]	.17 [.03, .31]	.08 [-.07, .22]	.36 [.23, .48]	.11 −.03, .25]	.19 [.04, .32]	.42 [.29, .53]	.41 [.28, .52]	.26 [.12, .39]	2.50	.012

ADHD = attention-deficit/hyperactivity disorder; CD = conduct disorder; CI = confidence interval; ODD = oppositional defiant disorder; ILF-EXTERNAL = Clinical Interview for Externalizing Disorders in Children and Adolescents.

^a^Due to floor effects, eight items that measured aggressive and antisocial behavior were excluded from the analyses.

## Discussion

This study evaluated the psychometric properties and factor structure of the DSM-5-based, semi-structured, clinical parent interview ILF-EXTERNAL using data from the ESCApreschool screening data set, which comprised preschool children aged 3–6-years screened for participation in the ESCApreschool multicenter study targeting preschool children with externalizing symptoms [38]. A notable strength of the ILF-EXTERNAL is its ability to provide both categorical and dimensional assessments.

In terms of the factor structure of the ILF-EXTERNAL, which has rarely been analyzed in previous studies using interviews, a first-order factor model with four correlated factors (Inattention, Hyperactivity-Impulsivity, ODD, CD) showed the best model fit and was straightforward to interpret. Notably, the intercorrelations between the factors in this model were fairly high, especially between the ODD and the CD factor both in the presented study (*r =* .76) and in a conceptually similar study with elementary school children [35; r = 0.75]. Despite this high correlation, 42% of the variance remained unshared, suggesting that the CD and ODD factors represent two closely related but distinct factors. The better fit of the first-order four-correlated-factor model compared to the unidimensional and the first-order two-factor model further supports this interpretation. Moreover, the consideration of CD and ODD as distinct dimensions aligns with their theoretical conceptualization: While ODD is primarily understood as a disorder of emotional regulation (irritability, anger, frustration tolerance), CD is a disorder characterized by serious antisocial and aggressive behavior [59,60]. Longitudinal data from the Great Smoky Mountains Study showed that although ODD in school age is a risk factor for later CD in boys, transitions from ODD to CD were less common than assumed [61]. In young adulthood, CD symptoms primarily predicted later delinquent and antisocial behavior, while ODD symptoms were more strongly associated with later anxiety and depressive disorders [61]. Population samples with a longitudinal design tended to show parallel, only partially interrelated developmental trajectories [61,62]. In summary, the amount of unshared variance, the superiority of the four-correlated-factor model over alternative models, and the alignment with theoretical considerations and empirical findings from previous studies suggest that ODD and CD, despite their high correlation and shared risk factors, represent two distinct, albeit closely related, disorders. In terms of the factor structure of ADHD, the presented findings correspond to previous literature and the DSM-5 / ICD-11, insofar as ADHD inattention and hyperactivity-impulsivity dimensions are considered as two different but related constructs [63]. On the whole, the item-factor configuration of the ILF-EXTERNAL was confirmed in a clinical sample of preschool children.

With regard to scale reliability, Cronbach’s alpha and McDonald’s omega coefficients for the ILF-EXTERNAL scales were generally acceptable to good, similarly to the findings for the DIPA scale [21]. An exception is the *CD Symptoms – Short Version* scale (α = .69, ω = .73), where Cronbach’s alpha fell just below the threshold of acceptance, but McDonald’s omega showed acceptable reliability. Similar internal consistencies of *CD Symptoms* scales have been reported by Frick, Barry [9] for the DISC-IV version 2.3 [α =  .59, 9] and in our own study examining psychometric properties of the ILF-EXTERNAL in school-age children [34]. We believe that this rather low internal consistency is unsurprising for several reasons: First, we excluded the items B08 to B15, which assess antisocial symptoms, as they are typically only relevant for older children. This resulted in a shorter scale comprising only seven items, with less variance. Second, the scores of the remaining items of this shortened scale showed a low mean (*M* = 0.44; *SD* = 0.43) and a skewed distribution. Third, the heterogeneity of these symptoms might have impaired the reliability of the scale [9]. While the *ADHD Functional Impairment* scale also showed low internal consistency (α = .65, ω = .68), which may likewise be explained by the small number of items and their heterogeneity, the *ODD/CD Functional Impairment* scale showed very good internal consistency despite comparable item heterogeneity (α = .84, ω = .73). It should be noted that the individual items of both scales capture distinct domains of functional impairment (family, preschool, etc.). Accordingly, only moderate inter-item correlations are to be expected and may be diagnostically informative. Furthermore, especially in preschool-aged children, developmental asynchrony across domains should be taken into account [e.g., 64]. With some exceptions, the item-total correlations were generally satisfactory. Interestingly, the exclusion of any of these items with item-total correlations below *r*_it_ = .30 did not lead to a noticeable change in the Cronbach’s alpha of the respective scales. Taken together, these findings indicate that the internal consistency of some ILF-EXTERNAL scales should be interpreted with caution and that further refinement and evaluation of individual scales in larger samples are warranted. Nevertheless, internal consistency represents only one aspect of reliability, and the present findings should be considered together with the evidence from interrater reliability (IRR) and validity analyses.

After calculating the ICC one-way random-effects model for single ICC(1,1) and average ICC(1,2) measurements, the ICC revealed “moderate” to “good” IRR in line with Koo and Li [53] and “fair” to “excellent” IRR in line with Cicchetti [55]. To the best of our knowledge, only one previous study has provided information on interrater reliabilities pertaining to semi-structured clinical interviews in preschool children. Gigengack, Hein [21] assessed IRR for categorical diagnoses in the seven DIPA modules using Fleiss’ κ for *n* = 14 randomly selected interviews, and further evaluated the continuous scores on these modules using ICC. The results indicated almost perfect IRR for ADHD inattentive type (ICC = .98), ADHD hyperactive-impulsive type (ICC = .98), ADHD combined type (ICC = .99), and ODD (ICC = .98). However, as it remains unclear which ICC model the authors calculated and whether they relied on single or average measurements, the ability to interpret their findings is limited. In our own study on the IRR of the ILF-EXTERNAL in school-age children [34], IRR values were predominantly very good to excellent. However, despite administering the same interview and using the same training procedures for interviewers, the IRR values in the present study are considerably lower than those reported in our previous study Thöne, Görtz-Dorten [34]. One possible explanation for these diverging results might lie in the challenge of differentiating between clinically relevant behavior and transient problem behavior within the normative range in preschool-aged children [e.g., 13,65,66]. For clinical practice, limited interrater agreements lead to the conclusion that diagnoses can indeed be made as early as preschool age, but that a particularly comprehensive and careful clinical assessment is necessary. There must be significant impairments in several areas of life and alternative explanations must be carefully ruled out [58].

Diagnostic agreement between the interviewers and the blind raters were moderate and was similar to, albeit somewhat lower than, the kappa agreements for externalizing disorders in the DIPA [21], the K-SADS [23], and the ILF-EXTERNAL in school-age children [34] as well as the ILF-EXTERNAL conducted online via video chat [37]. Interestingly, these findings reflect the categorial-dimensional debate [24,67] and the frequently reported lack of agreement between different clinicians [68–70]. The meta-analyses by Markon, Chmielewski [69], for instance, suggested an expected 15% increase in reliability and a 37% increase in validity when using dimensional rather than categorical measures of psychopathology.

With regard to convergent and divergent validity, our findings revealed moderate to strong correlations between the ILF-EXTERNAL scales and the German CBCL/1½−5 scales, which cover similar symptoms. Moreover, the ILF-EXTERNAL scales showed significantly stronger associations with the CBCL *Externalizing Problems* scale than with the *Internalizing Problems* scale, thus supporting construct validity. Furthermore, the ILF-EXTERNAL scales generally correlated more strongly with the corresponding CBCL scales than with the non-corresponding CBCL scales. As such, our results are largely consistent with previous research, which reported small to moderate associations between the CBCL and clinical diagnoses from semi-structured interviews to assess clinical symptoms in children and adolescents [23,34,71–73]. Importantly, we additionally included proxy rating forms (FBB-ADHS-V/SSV) to be completed by parents and preschool teachers, which cover the same DSM-5 symptoms as the ILF-EXTERNAL, uniquely allowing us to specifically compare ratings between clinicians, parents, and preschool teachers in a multi-source approach. The results revealed the highest bivariate correlations between clinicians and parents, followed by very weak to small correlations between clinicians and preschool teachers. On the one hand, the considerable correlations between parents and clinicians can be explained by the fact that the clinical assessment is based on an interview with the parents. On the other hand, while informant discrepancies were initially attributed to measurement error, invalidity, or rater bias [e.g., 74], there is growing recognition that children’s behavior varies considerably depending on the situation and that this contextual variability should be considered clinically significant information [e.g., 31]. Consistently, Overgaard and Oerbeck [63] demonstrated in a large-scale cohort study that considering ratings of preschool teachers alongside parent ratings at the age of 3 has the potential to increase the predictive validity of an ADHD diagnosis in boys at the age of 5. Two important clinical implications can be derived from these findings: First, since it is apparent that no single informant can fully capture the important variations in children’s functioning across different interaction partners and contexts, such as in preschool or at home, multi-informant assessment is particularly important [26,29,31,35,75–78]. Specifically, it seems advisable, in addition to a clinical interview with the parents, for example based on the ILF-EXTERNAL, to also explore the perspectives of (preschool) teachers, for example through questionnaires. Second, as the clinician is responsible for integrating these diverse informant assessments and forming his/her own clinical judgment, our results emphasize the crucial role of informed clinical judgment [cf. 35].

Several strengths and limitations of this study should be noted. A major strength lies in the systematic comparison of ratings by clinicians, parents, and teachers, thus applying a multi-source approach in a large multicenter study. A distinctive characteristic of the DISYPS-III is that all of the diagnostic instruments within a specific mental disorder domain share identical diagnostic scales, and largely consist of identical items, enabling an easy assessment and fostering a systematic comparison of different rater perspectives in a multi-informant approach [12].

With regard to limitations, it should be acknowledged that the analytical sample was derived from the ESCApreschool screening dataset [38], which comprised preschool children who were screened in the context of a multicenter study evaluating evidence-based stepped-care treatments for children with externalizing symptoms. Accordingly, the sample was enriched for externalizing symptoms and predominantly comprised children with ADHD (82.5%) and/or ODD (57.8%). Although all available screening cases were included, irrespective of whether children subsequently fulfilled the intervention eligibility criteria, the sample does not represent the full spectrum of externalizing behavior in the general preschool population. Nevertheless, the inclusion of all available screening cases resulted in a heterogeneous sample with variability in externalizing symptom severity and cognitive functioning beyond that represented by children fulfilling the ESCApreschool eligibility criteria. However, the relatively small sample size (*N* = 211 or *n* = 129) may further limit the generalizability of the findings. In terms of the factor structure of the ILF-EXTERNAL, while we endeavored to incorporate the symptoms from the *DMDD* scale and the *LPE* scale into our CFAs, this was not possible due to the rather small sample size (*N* = 211). Furthermore, the small sample size of the female subgroup did not allow for an investigation of measurement invariance across gender. A further limiting factor is that we had to exclude eight items from the CD scale analysis due to substantial floor effects, which is in line with prevalence rates and considerations of developmental psychology. The excluded items assessed more severe forms of antisocial behavior and their removal resulted in a limited coverage of the DSM-5 CD construct. Consequently, findings related to the CD scale should be interpreted with caution, as the retained items may not fully capture the breadth of CD symptoms, thereby limiting the comparability and interpretability of the findings. Furthermore, we tested a higher-order factor model with two second-order factors (ADHD and disruptive behavior symptoms) but found no convergence. Accordingly, future research should include more externalizing symptoms and potentially also functional impairment domains, as well as larger samples, in order to further explore the hierarchical nature of the externalizing spectrum [cf., 79] and examine possible gender differences.

Overall, the present findings should be considered as initial evidence supporting the reliability and validity of the ILF-EXTERNAL. Further studies are needed to replicate these findings in independent samples and to extend the psychometric evaluation of the instrument, including additional aspects of validity and clinical utility.

## Conclusions

The present study provided a comprehensive psychometric evaluation of the clinical parent interview for diagnosing externalizing disorders in children and adolescents (ILF-EXTERNAL) in clinically referred preschool children. More generally, the study contributes to the debate about categorical versus dimensional concepts of diagnostic classification, through a consideration of both. Overall, we were able to confirm the a priori assumed DSM-5-based factor structure of the ILF-EXTERNAL, as demonstrated by the correlated factors model with four factors (Inattention, Hyperactivity-Impulsivity, ODD, CD). With regard to categorical vs. dimensional agreement, IRR estimates at the dimensional level were generally higher than those at the categorical level. This highlights that mental disorders are not characterized by abrupt thresholds and are rather better described as continuous phenomena. Our findings provide evidence for the convergent and divergent validity of the ILF-EXTERNAL, as indicated by moderate to high correlations between the ILF-EXTERNAL scale scores and corresponding externalizing symptom scales, and by significantly lower correlations with divergent symptom scales from the CBCL/1½−5. This study revealed a complex picture of how associations between different informants can vary depending on the context (e.g., preschool vs. home), highlighting the need to systematically integrate multiple informants into clinical decision making. In conclusion, the present findings provide preliminary evidence that the ILF-EXTERNAL is a factorially sound, reliable, and valid semi-structured clinical interview for the categorical and dimensional assessment of externalizing disorders in both clinical practice and research.

## Supporting information

S1 Table Violated inclusion and exclusion criteria in screening negatives of the ESCApreschool Study (*n* = 21). Four children violate two inclusion or exclusion criteria. For two children listed as screening negatives, it is unclear which of the inclusion and exclusion criteria have been violated (missing values). For two children, reasons other than those mentioned were decisive for their non-inclusion in the ESCApreschool study.
(PDF)

S1 Fig Standardized factor loadings and covariance structure of the unidimensional CFA. Factor descriptions: esd = Externalizing Spectrum Disorders. Item descriptions: ad_a1 = *Careless*; ad_a2 = *Difficulties Sustaining Attention*; ad_a3 = *Does not Listen*; ad_a4 = *Does not Finish Work*; ad_a5 = *Organizational Skills*; ad_a6 = *Concentration*; ad_a7 = *Loses Things*; ad_a8 = *Easily Distracted*; ad_a9 = *Forgetful*; ad_b1 = *Fidgets*; ad_b2 = *Leaves Seat*; ad_b3 = *Runs, Climbs*; ad_b4 = *Difficulties Playing Quietly*; ad_b5 = *Driven, on the go*; ad_b6 = *Blurts out Answers*; ad_b7 = *Waits for Turn*; ad_b8 = *Interrupts, Intrudes*; ad_b9 = *Talks Excessively*; ss_a1 = *Loses Temper*; ss_a2 = *Touchy, Easily Annoyed*; ss_a3 = *Angry, Resentful*; ss_a4 = *Argues with Adults*; ss_a5 = *Refuses to Comply with Requests*; ss_a6 = *Annoys*; ss_a7 = *Blames Others*; ss_a8 = *Spiteful, Vindictive*; ss_b1 = *Physical Fights*; ss_b2 = *Bullies, Threatens, or Intimidates*; ss_b3 = *Cruel to Animals*; ss_b4 = *Lies*; ss_b5 = *Steals without Confrontation;* ss_b6 = *Uses Weapons in Fight*; ss_b7 = *Cruel to People*. Due to floor effects, eight items that measured aggressive and antisocial behavior were excluded from the analyses. *p < .05, ** *p* < .01, ***p < .001.
(TIFF)

S2 Fig Standardized factor loadings and covariance structure of the first-order CFA with two correlated factors. Factor descriptions: adhd = Attention-Deficit/Hyperactivity Disorder; ssv = Disruptive Behavior Disorders (oppositional defiant disorder and conduct disorder combined). Item descriptions: ad_a1 = Careless; ad_a2 = Difficulties Sustaining Attention; ad_a3 = Does not Listen; ad_a4 = Does not Finish Work; ad_a5 = Organizational Skills; ad_a6 = Concentration; ad_a7 = Loses Things; ad_a8 = Easily Distracted; ad_a9 = Forgetful; ad_b1 = Fidgets; ad_b2 = Leaves Seat; ad_b3 = Runs, Climbs; ad_b4 = Difficulties Playing Quietly; ad_b5 = Driven, on the go; ad_b6 = Blurts out Answers; ad_b7 = Waits for Turn; ad_b8 = Interrupts, Intrudes; ad_b9 = Talks Excessively; ss_a1 = Loses Temper; ss_a2 = Touchy, Easily Annoyed; ss_a3 = Angry, Resentful; ss_a4 = Argues with Adults; ss_a5 = Refuses to Comply with Requests; ss_a6 = Annoys; ss_a7 = Blames Others; ss_a8 = Spiteful, Vindictive; ss_b1 = Physical Fights; ss_b2 = Bullies, Threatens, or Intimidates; ss_b3 = Cruel to Animals; ss_b4 = Lies; ss_b5 = Steals without Confrontation; ss_b6 = Uses Weapons in Fight; ss_b7 = Cruel to People. Due to floor effects, eight items that measured aggressive and antisocial behavior were excluded from the analyses. *p < .05, ** p < .01, ***p < .001.
(TIFF)

S3 Fig Standardized factor loadings and covariance structure of the second-order CFA. Factor descriptions: esd = Externalizing Spectrum Disorders; ina = Inattention; hi = Hyperactivity-impulsivity; odd = Oppositional Defiant Disorder; cd = Conduct Disorder. Item descriptions: ad_a1 = *Careless*; ad_a2 = *Difficulties Sustaining Attention*; ad_a3 = *Does not Listen*; ad_a4 = *Does not Finish Work*; ad_a5 = *Organizational Skills*; ad_a6 = *Concentration*; ad_a7 = *Loses Things*; ad_a8 = *Easily Distracted*; ad_a9 = *Forgetful*; ad_b1 = *Fidgets*; ad_b2 = *Leaves Seat*; ad_b3 = *Runs, Climbs*; ad_b4 = *Difficulties Playing Quietly*; ad_b5 = *Driven, on the go*; ad_b6 = *Blurts out Answers*; ad_b7 = *Waits for Turn*; ad_b8 = *Interrupts, Intrudes*; ad_b9 = *Talks Excessively*; ss_a1 = *Loses Temper*; ss_a2 = *Touchy, Easily Annoyed*; ss_a3 = *Angry, Resentful*; ss_a4 = *Argues with Adults*; ss_a5 = *Refuses to Comply with Requests*; ss_a6 = *Annoys*; ss_a7 = *Blames Others*; ss_a8 = *Spiteful, Vindictive*; ss_b1 = *Physical Fights*; ss_b2 = *Bullies, Threatens, or Intimidates*; ss_b3 = *Cruel to Animals*; ss_b4 = *Lies*; ss_b5 = *Steals without Confrontation;* ss_b6 = *Uses Weapons in Fight*; ss_b7 = *Cruel to People*. Due to floor effects, eight items that measured aggressive and antisocial behavior were excluded from the analyses. *p < .05, ** *p* < .01, ***p < .001.
(TIFF)

S4 Fig Standardized factor loadings and covariance structure of the bifactor CFA. Factor descriptions: esd = Externalizing Spectrum Disorders; ina = Inattention; hi = Hyperactivity-Impulsivity; odd = Oppositional Defiant Disorder; cd = Conduct Disorder. Item descriptions: ad_a1 = *Careless*; ad_a2 = *Difficulties Sustaining Attention*; ad_a3 = *Does not Listen*; ad_a4 = *Does not Finish Work*; ad_a5 = *Organizational Skills*; ad_a6 = *Concentration*; ad_a7 = *Loses Things*; ad_a8 = *Easily Distracted*; ad_a9 = *Forgetful*; ad_b1 = *Fidgets*; ad_b2 = *Leaves Seat*; ad_b3 = *Runs, Climbs*; ad_b4 = *Difficulties Playing Quietly*; ad_b5 = *Driven, on the go*; ad_b6 = *Blurts out Answers*; ad_b7 = *Waits for Turn*; ad_b8 = *Interrupts, Intrudes*; ad_b9 = *Talks Excessively*; ss_a1 = *Loses Temper*; ss_a2 = *Touchy, Easily Annoyed*; ss_a3 = *Angry, Resentful*; ss_a4 = *Argues with Adults*; ss_a5 = *Refuses to Comply with Requests*; ss_a6 = *Annoys*; ss_a7 = *Blames Others*; ss_a8 = *Spiteful, Vindictive*; ss_b1 = *Physical Fights*; ss_b2 = *Bullies, Threatens, or Intimidates*; ss_b3 = *Cruel to Animals*; ss_b4 = *Lies*; ss_b5 = *Steals without Confrontation;* ss_b6 = *Uses Weapons in Fight*; ss_b7 = *Cruel to People*. Due to floor effects, eight items that measured aggressive and antisocial behavior were excluded from the analyses. *p < .05, ** *p* < .01, ***p < .001.
(TIFF)

## References

[1] Task Force on Research Diagnostic Criteria: Infancy Preschool. Research diagnostic criteria for infants and preschool children: the process and empirical support. J Am Acad Child Adolesc Psychiatry. 2003;42(12):1504–12. doi: 10.1097/01.chi.0000091504.46853.0a 14627886

[2] Bufferd SJ, Dougherty LR, Carlson GA, Rose S, Klein DN. Psychiatric disorders in preschoolers: continuity from ages 3 to 6. Am J Psychiatry. 2012;169(11):1157–64. doi: 10.1176/appi.ajp.2012.12020268 23128922 PMC3513401

[3] Roza SJ, Hofstra MB, van der Ende J, Verhulst FC. Stable prediction of mood and anxiety disorders based on behavioral and emotional problems in childhood: a 14-year follow-up during childhood, adolescence, and young adulthood. Am J Psychiatry. 2003;160(12):2116–21. doi: 10.1176/appi.ajp.160.12.2116 14638580

[4] Lahey BB, Lee SS, Sibley MH, Applegate B, Molina BSG, Pelham WE. Predictors of adolescent outcomes among 4-6-year-old children with attention-deficit/hyperactivity disorder. J Abnorm Psychol. 2016;125(2):168–81. doi: 10.1037/abn0000086 26854503 PMC4747048

[5] Mulraney M, Coghill D, Bishop C, Mehmed Y, Sciberras E, Sawyer M, et al. A systematic review of the persistence of childhood mental health problems into adulthood. Neurosci Biobehav Rev. 2021;129:182–205. doi: 10.1016/j.neubiorev.2021.07.030 34363845

[6] Rettew DC, Lynch AD, Achenbach TM, Dumenci L, Ivanova MY. Meta-analyses of agreement between diagnoses made from clinical evaluations and standardized diagnostic interviews. Int J Methods Psychiatr Res. 2009;18(3):169–84. doi: 10.1002/mpr.289 19701924 PMC6878243

[7] Hoyer J, Knappe S. Psychotherapie braucht strukturierte Diagnostik!. PiD - Psychotherapie im Dialog. 2012;13(01):2–5.

[8] Nordgaard J, Sass LA, Parnas J. The psychiatric interview: validity, structure, and subjectivity. Eur Arch Psychiatry Clin Neurosci. 2013;263(4):353–64. doi: 10.1007/s00406-012-0366-z 23001456 PMC3668119

[9] Frick PJ, Barry CT, Kamphaus RW. Clinical assessment of child and adolescent personality and behavior. 3rd ed. New York, NY: Springer Science Business Media. 2010.

[10] Leffler JM, Riebel J, Hughes HM. A Review of Child and Adolescent Diagnostic Interviews for Clinical Practitioners. Assessment. 2015;22(6):690–703. doi: 10.1177/1073191114561253 25520212

[11] Segal DL, Williams KN. Structured and semistructured interviews for differential diagnosis: fundamental issues, applications, and features. In: Beidel DC, Frueh BC, Hersen M, editors. Adult psychopathology and diagnosis. 7th ed. Hoboken, NJ: John Wiley & Sons, Inc. 2014. 103–29.

[12] Döpfner M, Görtz-Dorten A, Petermann F. Diagnostik psychischer Störungen im Kindes- und Jugendalter. 4th ed. Göttingen: Hogrefe. 2023.

[13] Egger HL, Angold A. Common emotional and behavioral disorders in preschool children: presentation, nosology, and epidemiology. J Child Psychol Psychiatry. 2006;47(3–4):313–37. doi: 10.1111/j.1469-7610.2006.01618.x 16492262

[14] Shaffer D, Fisher P, Lucas CP, Dulcan MK, Schwab-Stone ME. NIMH Diagnostic Interview Schedule for Children Version IV (NIMH DISC-IV): description, differences from previous versions, and reliability of some common diagnoses. J Am Acad Child Adolesc Psychiatry. 2000;39(1):28–38. doi: 10.1097/00004583-200001000-00014 10638065

[15] American Psychiatric Association, Work Group on D-I-P. Diagnostic and statistical manual of mental disorders, fourth edition: primary care version. 1st ed. Washington, DC: American Psychiatric Association. 1995.

[16] Luby JL, Heffelfinger AK, Mrakotsky C, Hessler MJ, Brown KM, Hildebrand T. Preschool major depressive disorder: preliminary validation for developmentally modified DSM-IV criteria. J Am Acad Child Adolesc Psychiatry. 2002;41(8):928–37. doi: 10.1097/00004583-200208000-00011 12162628

[17] Egger HL, Angold A, Small B, Copeland W. The preschool age psychiatric assessment: a structured parent interview for assessing psychiatric symptoms and disorders in preschool children. In: DelCarmen-Wiggins R, Carter AS, editors. Handbook of infant, toddler, and preschool mental health assessment. 2 ed. New York, NY: Oxford University Press. 2019. 226–43.

[18] Angold A, Costello EJ. The Child and Adolescent Psychiatric Assessment (CAPA). J Am Acad Child Adolesc Psychiatry. 2000;39(1):39–48. doi: 10.1097/00004583-200001000-00015 10638066

[19] Egger HL, Erkanli A, Keeler G, Potts E, Walter BK, Angold A. Test-Retest Reliability of the Preschool Age Psychiatric Assessment (PAPA). J Am Acad Child Adolesc Psychiatry. 2006;45(5):538–49. doi: 10.1097/01.chi.0000205705.71194.b8 16601400

[20] Scheeringa MS, Haslett N. The reliability and criterion validity of the Diagnostic Infant and Preschool Assessment: a new diagnostic instrument for young children. Child Psychiatry Hum Dev. 2010;41(3):299–312. doi: 10.1007/s10578-009-0169-2 20052532 PMC2862973

[21] Gigengack MR, Hein IM, van Meijel EPM, Lindeboom R, van Goudoever JB, Lindauer RJL. Accuracy of the Diagnostic Infant and Preschool Assessment (DIPA) in a Dutch sample. Compr Psychiatry. 2020;100:152177. doi: 10.1016/j.comppsych.2020.152177 32360141

[22] Kaufman J, Birmaher B, Brent D, Rao U, Flynn C, Moreci P, et al. Schedule for Affective Disorders and Schizophrenia for School-Age Children-Present and Lifetime Version (K-SADS-PL): initial reliability and validity data. J Am Acad Child Adolesc Psychiatry. 1997;36(7):980–8. doi: 10.1097/00004583-199707000-00021 9204677

[23] Birmaher B, Ehmann M, Axelson DA, Goldstein BI, Monk K, Kalas C, et al. Schedule for affective disorders and schizophrenia for school-age children (K-SADS-PL) for the assessment of preschool children--a preliminary psychometric study. J Psychiatr Res. 2009;43(7):680–6. doi: 10.1016/j.jpsychires.2008.10.003 19000625 PMC2736874

[24] Coghill D, Sonuga-Barke EJS. Annual research review: categories versus dimensions in the classification and conceptualisation of child and adolescent mental disorders--implications of recent empirical study. J Child Psychol Psychiatry. 2012;53(5):469–89. doi: 10.1111/j.1469-7610.2011.02511.x 22288576

[25] Achenbach TM, Rescorla LA. Manual for the ASEBA Preschool Forms & Profiles. Burlington, VT: University of Vermont, Research Center for Children, Youth and Families. 2000.

[26] Achenbach TM. Bottom-Up and Top-Down Paradigms for Psychopathology: A Half-Century Odyssey. Annu Rev Clin Psychol. 2020;16:1–24. doi: 10.1146/annurev-clinpsy-071119-115831 32383999

[27] Palermo TM, Long AC, Lewandowski AS, Drotar D, Quittner AL, Walker LS. Evidence-based assessment of health-related quality of life and functional impairment in pediatric psychology. J Pediatr Psychol. 2008;33(9):983–96; discussion 997-8. doi: 10.1093/jpepsy/jsn038 18430762 PMC2543105

[28] Rapee RM, Bőgels SM, van der Sluis CM, Craske MG, Ollendick T. Annual research review: conceptualising functional impairment in children and adolescents. J Child Psychol Psychiatry. 2012;53(5):454–68. doi: 10.1111/j.1469-7610.2011.02479.x 22067073

[29] De Los Reyes A, Thomas SA, Goodman KL, Kundey SMA. Principles underlying the use of multiple informants’ reports. Annu Rev Clin Psychol. 2013;9:123–49. doi: 10.1146/annurev-clinpsy-050212-185617 23140332 PMC4103654

[30] De Los Reyes A, Augenstein TM, Wang M, Thomas SA, Drabick DAG, Burgers DE, et al. The validity of the multi-informant approach to assessing child and adolescent mental health. Psychol Bull. 2015;141(4):858–900. doi: 10.1037/a0038498 25915035 PMC4486608

[31] Dirks MA, De Los Reyes A, Briggs-Gowan M, Cella D, Wakschlag LS. Annual research review: embracing not erasing contextual variability in children’s behavior--theory and utility in the selection and use of methods and informants in developmental psychopathology. J Child Psychol Psychiatry. 2012;53(5):558–74. doi: 10.1111/j.1469-7610.2012.02537.x 22360546 PMC4720148

[32] Görtz-Dorten A, Thöne AK, Döpfner M. DISYPS-ILF: Interview-Leitfäden zum Diagnostik-System für psychische Störungen nach DSM-5 für Kinder- und Jugendliche. Göttingen: Hogrefe. 2022.

[33] Döpfner M, Görtz-Dorten A. Diagnostik-System für psychische Störungen nach ICD-10 und DSM-5 für Kinder und Jugendliche - III (DISYPS-III). Göttingen: Hogrefe. 2017.

[34] Thöne A-K, Görtz-Dorten A, Altenberger P, Dose C, Geldermann N, Hautmann C, et al. Toward a Dimensional Assessment of Externalizing Disorders in Children: Reliability and Validity of a Semi-Structured Parent Interview. Front Psychol. 2020;11:1840. doi: 10.3389/fpsyg.2020.01840 32849082 PMC7396521

[35] Thöne A-K, Junghänel M, Görtz-Dorten A, Dose C, Hautmann C, Jendreizik LT, et al. Disentangling symptoms of externalizing disorders in children using multiple measures and informants. Psychol Assess. 2021;33(11):1065–79. doi: 10.1037/pas0001053 34435849

[36] Thöne AK, Dose C, Junghänel M, Hautmann C, Jendreizik LT, Treier AK. Identifying symptoms of ADHD and disruptive behavior disorders most strongly associated with functional impairment in children: a symptom-level approach. J Psychopathol Behav Assess. 2023;45(2):277–93.

[37] Weyrich S, Scholz V, Hetzke L, Ulsamer S, Wallau C, Mager D, et al. Psychometric evaluation of the Clinical Interview for Externalizing Disorders (ILF-EXTERNAL) in an online setting - Results from the consortium project INTEGRATE-ADHD. J Health Monit. 2024;9(3):e12536. doi: 10.25646/12536 39411330 PMC11459213

[38] Becker K, Banaschewski T, Brandeis D, Dose C, Hautmann C, Holtmann M, et al. Individualised stepwise adaptive treatment for 3-6-year-old preschool children impaired by attention-deficit/hyperactivity disorder (ESCApreschool): study protocol of an adaptive intervention study including two randomised controlled trials within the consortium ESCAlife. Trials. 2020;21(1):56. doi: 10.1186/s13063-019-3872-8 31918739 PMC6953462

[39] Breuer D, Döpfner M. Entwicklung eines Fragebogens zur Erfassung von Aufmerksamkeitsdefizit-/Hyperaktivitätsstörungen (ADHS) bei Vorschulkindern im Eltern- und im Erziehungsurteil. Z Entwicklungspsychol Padagog Psychol. 2008;40(1):40–8.

[40] Görtz-Dorten A, Ise E, Hautmann C, Walter D, Döpfner M. Psychometric properties of a German parent rating scale for oppositional defiant and conduct disorder (FBB-SSV) in clinical and community samples. Child Psychiatry Hum Dev. 2014;45(4):388–97. doi: 10.1007/s10578-013-0409-3 24126739

[41] Korsch F, Petermann F. Agreement between parents and teachers on preschool children’s behavior in a clinical sample with externalizing behavioral problems. Child Psychiatry Hum Dev. 2014;45(5):617–27. doi: 10.1007/s10578-013-0430-6 24363143

[42] Plück J, Scholz K-K, Döpfner M. CBCL/1½-5, C-TRF/1½-5. Deutsche Kleinkind- und Vorschulalter-Formen der Child Behavior Checklist von Thomas M. Achenbach und Leslie A. Rescorla. Göttingen: Hogrefe. 2022.

[43] Ehmke T, Siegle T. ISEI, ISCED, HOMEPOS, ESCS: Indikatoren der sozialen Herkunft bei der Quantifizierung von sozialen Disparitäten. Z Erziehwiss. 2005;8(4):521–39.

[44] R Core Team. R: A language and environment for statistical computing. Vienna, Austria: R Foundation for Statistical Computing. 2022.

[45] Bennett DA. How can I deal with missing data in my study?. Aust N Z J Public Health. 2001;25(5):464–9. doi: 10.1111/j.1467-842x.2001.tb00294.x 11688629

[46] Hooper D, Coughlan J, Mullen MR. Structural equation modelling: guidelines for determining model fit. Electronic Journal of Business Research Methods. 2008;6(1):53–60.

[47] Hu L, Bentler PM. Cutoff criteria for fit indexes in covariance structure analysis: Conventional criteria versus new alternatives. Structural Equation Modeling: A Multidisciplinary Journal. 1999;6(1):1–55. doi: 10.1080/10705519909540118

[48] Nunnally JC. Psychometric theory. New York, NY: McGraw-Hill. 1967.

[49] Field A. Discovering Statistics Using IBM SPSS Statistics. 5th ed. London, England: SAGE Publications. 2018.

[50] McGraw KO, Wong SP. Forming inferences about some intraclass correlation coefficients. Psychological Methods. 1996;1(1):30–46. doi: 10.1037/1082-989x.1.1.30

[51] Shrout PE, Fleiss JL. Intraclass correlations: uses in assessing rater reliability. Psychol Bull. 1979;86(2):420–8. doi: 10.1037/0033-2909.86.2.420 18839484

[52] Hallgren KA. Computing Inter-Rater Reliability for Observational Data: An Overview and Tutorial. Tutor Quant Methods Psychol. 2012;8(1):23–34. doi: 10.20982/tqmp.08.1.p023 22833776 PMC3402032

[53] Koo TK, Li MY. A Guideline of Selecting and Reporting Intraclass Correlation Coefficients for Reliability Research. J Chiropr Med. 2016;15(2):155–63. doi: 10.1016/j.jcm.2016.02.012 27330520 PMC4913118

[54] LeBreton JM, Senter JL. Answers to 20 Questions About Interrater Reliability and Interrater Agreement. Organizational Research Methods. 2007;11(4):815–52. doi: 10.1177/1094428106296642

[55] Cicchetti DV. Guidelines, criteria, and rules of thumb for evaluating normed and standardized assessment instruments in psychology. Psychol Assess. 1994;6(4):284–90.

[56] Cohen J. A coefficient of agreement for nominal scales. Educ Psychol Meas. 1960;20(1):37–46.

[57] Landis JR, Koch GG. The measurement of observer agreement for categorical data. Biometrics. 1977;33(1):159–74. doi: 10.2307/2529310 843571

[58] Steiger JH. Tests for comparing elements of a correlation matrix. Psychological Bulletin. 1980;87(2):245–51.

[59] Cavanagh M, Quinn D, Duncan D, Graham T, Balbuena L. Oppositional Defiant Disorder Is Better Conceptualized as a Disorder of Emotional Regulation. J Atten Disord. 2017;21(5):381–9. doi: 10.1177/1087054713520221 24481934

[60] Burke JD, Evans SC, Carlson GA. Debate: Oppositional defiant disorder is a real disorder. Child Adolesc Ment Health. 2022;27(3):297–9. doi: 10.1111/camh.12588 35869580

[61] Rowe R, Costello EJ, Angold A, Copeland WE, Maughan B. Developmental pathways in oppositional defiant disorder and conduct disorder. J Abnorm Psychol. 2010;119(4):726–38. doi: 10.1037/a0020798 21090876 PMC3057683

[62] Diamantopoulou S, Verhulst FC, van der Ende J. The parallel development of ODD and CD symptoms from early childhood to adolescence. Eur Child Adolesc Psychiatry. 2011;20(6):301–9. doi: 10.1007/s00787-011-0175-3 21499848 PMC3098986

[63] Willcutt EG, Nigg JT, Pennington BF, Solanto MV, Rohde LA, Tannock R, et al. Validity of DSM-IV attention deficit/hyperactivity disorder symptom dimensions and subtypes. J Abnorm Psychol. 2012;121(4):991–1010. doi: 10.1037/a0027347 22612200 PMC3622557

[64] Overgaard KR, Oerbeck B, Friis S, Pripp AH, Aase H, Ingeborgrud CB, et al. Functional Impairment Related to ADHD From Preschool to School Age. J Atten Disord. 2025;29(3):220–30. doi: 10.1177/10870547241301179 39625105 PMC11694549

[65] Overgaard KR, Oerbeck B, Friis S, Pripp AH, Aase H, Biele G, et al. Attention-deficit/hyperactivity disorder from preschool to school age: change and stability of parent and teacher reports. Eur Child Adolesc Psychiatry. 2023;32(10):1947–55. doi: 10.1007/s00787-022-02019-1 35737107 PMC10533600

[66] Overgaard KR, Oerbeck B, Friis S, Pripp AH, Aase H, Zeiner P. Predictive validity of attention-deficit/hyperactivity disorder from ages 3 to 5 Years. Eur Child Adolesc Psychiatry. 2022;31(7):1–10. doi: 10.1007/s00787-021-01750-5 33677627 PMC9343262

[67] Clark LA, Cuthbert B, Lewis-Fernández R, Narrow WE, Reed GM. Three Approaches to Understanding and Classifying Mental Disorder: ICD-11, DSM-5, and the National Institute of Mental Health’s Research Domain Criteria (RDoC). Psychol Sci Public Interest. 2017;18(2):72–145. doi: 10.1177/1529100617727266 29211974

[68] Chmielewski M, Clark LA, Bagby RM, Watson D. Method matters: Understanding diagnostic reliability in DSM-IV and DSM-5. J Abnorm Psychol. 2015;124(3):764–9. doi: 10.1037/abn0000069 26098046 PMC4573819

[69] Markon KE, Chmielewski M, Miller CJ. The reliability and validity of discrete and continuous measures of psychopathology: a quantitative review. Psychol Bull. 2011;137(5):856–79. doi: 10.1037/a0023678 21574681

[70] Regier DA, Narrow WE, Clarke DE, Kraemer HC, Kuramoto SJ, Kuhl EA, et al. DSM-5 field trials in the United States and Canada, Part II: test-retest reliability of selected categorical diagnoses. Am J Psychiatry. 2013;170(1):59–70. doi: 10.1176/appi.ajp.2012.12070999 23111466

[71] Kim YS, Cheon KA, Kim BN, Chang SA, Yoo HJ, Kim JW. The reliability and validity of Kiddie-Schedule for Affective Disorders and Schizophrenia-Present and Lifetime Version - Korean version (K-SADS-PL-K). Yonsei Med J. 2004;45(1):81–9. 15004873 10.3349/ymj.2004.45.1.81

[72] Brasil HHA, Bordin IA. Convergent validity of K-SADS-PL by comparison with CBCL in a Portuguese speaking outpatient population. BMC Psychiatry. 2010;10:83. doi: 10.1186/1471-244X-10-83 20955616 PMC2984471

[73] Chen Y-L, Shen L-J, Gau SS-F. The Mandarin version of the Kiddie-Schedule for Affective Disorders and Schizophrenia-Epidemiological version for DSM-5 - A psychometric study. J Formos Med Assoc. 2017;116(9):671–8. doi: 10.1016/j.jfma.2017.06.013 28709821

[74] Roberts BW, Caspi A. Personality development and the person–situation debate: it’s déjà vu all over again. Psychol Inq. 2001;12(2):104–9.

[75] De Los Reyes A. Introduction to the special section: More than measurement error: Discovering meaning behind informant discrepancies in clinical assessments of children and adolescents. J Clin Child Adolesc Psychol. 2011;40(1):1–9. doi: 10.1080/15374416.2011.533405 21229439

[76] De Los Reyes A, Epkins CC. Introduction to the Special Issue. A Dozen Years of Demonstrating That Informant Discrepancies are More Than Measurement Error: Toward Guidelines for Integrating Data from Multi-Informant Assessments of Youth Mental Health. J Clin Child Adolesc Psychol. 2023;52(1):1–18. doi: 10.1080/15374416.2022.2158843 36725326

[77] Hunsley J, Mash EJ. Evidence-based assessment. Annu Rev Clin Psychol. 2007;3:29–51. doi: 10.1146/annurev.clinpsy.3.022806.091419 17716047

[78] Thöne AK, Junghänel M, Görtz-Dorten A, Breuer D, del Giudice T, Hanisch C. Empirically based dimensions of externalizing symptoms in children and adolescents: A multitrait-multisource approach. J Psychopathol Behav Assess. 2022;44(3):844–61.

[79] Krueger RF, Hobbs KA, Conway CC, Dick DM, Dretsch MN, Eaton NR, et al. Validity and utility of Hierarchical Taxonomy of Psychopathology (HiTOP): II. Externalizing superspectrum. World Psychiatry. 2021;20(2):171–93. doi: 10.1002/wps.20844 34002506 PMC8129870

