# Supplementary material for: Psychometric evaluation of a DSM-5-based semi-structured parent interview for externalizing disorders in preschool children
Source: PLoS One. 2026 Sep 15;21(9):e0357785. doi: 10.1371/journal.pone.0357785 (PMC13577363; doi:10.1371/journal.pone.0357785)
Supplement: Supplementary file 1 [file pone.0357785.s001.pdf]

Violated inclusion criteria	*n* (%)
(1) age between 3;0 and 6;11 years	0 (0.0)
(2) attending preschool	1 (5.3)
(3) exhibiting externalizing behavior problems	10 (52.6)
(4) informed consent / assent to participate in the intervention study	1 (5.3)
Violated exclusion criteria	*n* (%)
(1) child’s IQ < 80	8 (44.4)
(2) diagnosis of pervasive developmental disorder, schizophrenia, bipolar disorder, or severe depressive episode	1 (5.9)
(3) insufficient parental German-language skills	0 (0.0)
(4) psychotropic medication of the child (except for ADHD medication)	0 (0.0)
(5) child’s participation in a regular intensive behavior therapy.	0 (0.0)
